# Improved extracellular vesicle-based mRNA delivery for familial hypercholesterolemia treatment

**DOI:** 10.7150/thno.82873

**Published:** 2023-06-12

**Authors:** Zheng Yang, Panpan Ji, Zhelong Li, Rongxin Zhang, Mengying Wei, Yang Yang, Lijun Yuan, Yan Han, Guodong Yang

**Affiliations:** 1 College of Otolaryngology, Head and Neck Surgery, Chinese PLA General Hospital, Beijing 100853, China.; 2 Department of Plastic Surgery, Chinese PLA General Hospital, Beijing, China.; 3 The State Laboratory of Cancer Biology, Department of Biochemistry and Molecular Biology, Fourth Military Medical University, Xi'an, Shaanxi, China.; 4 The State Laboratory of Cancer Biology, Department of Gastrointestinal Surgery, Xijing Hospital of Digestive Diseases, Fourth Military Medical University, Xi'an, Shaanxi, China.; 5 Department of Ultrasound Diagnostics, Tangdu Hospital, Fourth Military Medical University, Xi'an, Shaanxi, China.; 6 College of Life Sciences, Northwest University, Xi'an, Shaanxi, China.; 7 Department of Plastic Surgery, Xi'an Daxing Hospital, Xi'an, Shaanxi, China.

**Keywords:** Extracellular vesicles, therapeutic mRNA, RNA aptamer, loading efficiency, endosome escape, low-density lipoprotein receptor

## Abstract

Extracellular vesicle (EV)-based low-density lipoprotein receptor (*Ldlr*) mRNA delivery showed excellent therapeutic effects in treating familial hypercholesterolemia (FH). Nevertheless, the loading inefficiency of EV-based mRNA delivery presents a significant challenge. Recently, RNA-binding proteins (RBPs) have been fused to EV membrane proteins for selectively encapsulating targeted RNAs to promote loading efficiency. However, the strong interaction between therapeutic RNAs and RBPs prevents RNA release from endosomes to the cytosol in the recipient cells. In this study, an improved strategy was developed for efficient encapsulation of *Ldlr* mRNA into EVs in donor cells and controllable release in recipient cells.

**Methods:** The MS2 bacteriophage coat protein (CD9-MCP) fusion protein, *Ldlr* mRNA, and a customized MS2 containing RNA aptamer base-pair matched with *Ldlr* mRNA were expressed in donor cells. Cells receiving the above therapeutic EVs were simultaneously treated with EVs containing “*Ldlr* releaser” with a sequence similar to the recognition sites in *Ldlr* mRNA. Therapeutic effects were analyzed in *Ldlr*^-/-^ mice receiving EV treatments via the tail vein.

**Results:** *In vitro* experiments demonstrated improved loading efficiency of *Ldlr* mRNA in EVs via MS2-MCP interaction. Treatment of “*Ldlr* releaser” competitively interacted with MS2 aptamer with higher affinity and released *Ldlr* mRNA from CD9-MCP for efficient translation. When the combinatory EVs were delivered into recipient hepatocytes, the robust LDLR expression afforded therapeutic benefits in *Ldlr*^-/-^ mice.

**Conclusion:** We proposed an EV-based mRNA delivery strategy for enhanced encapsulation of therapeutic mRNAs in EVs and RNA release into the cytosol for translation in recipient cells with great potential for gene therapy.

## Introduction

Familial hypercholesterolemia (FH), characterized by a severe increase in plasma low-density lipoprotein-cholesterol (LDL-C) and premature coronary heart disease, is mainly caused by Low-density lipoprotein receptor (LDLR) dysfunction^1^-^3^. LDLR is a crucial lipoprotein receptor on the hepatocyte surface, essential for LDL-C clearance 4, 5. Increasing evidence confirmed that restoring LDLR expression in the liver could efficiently decrease total cholesterol 6-8. Extracellular vesicles (EVs) are lipid-bound vesicles secreted by cells into the extracellular space and have recently emerged as a promising vehicle for nucleic acid-based therapeutics in gene therapy 9. EVs possess multiple advantages for the delivery of therapeutics, such as easy manipulation, high biological penetration, and low immunogenicity 10-13. Our recent study verified the excellent therapeutic effects of EV-based *Ldlr* mRNA delivery for FH treatment. *Ldlr* mRNA was stably encapsulated in EVs and delivered into recipient cells, restoring *Ldlr* expression in the *Ldlr*^-/-^ mouse model 14. However, the loading inefficiency, especially for large-size mRNAs, limits the application of EV-based mRNA delivery 15-18.

Several studies have shown that EVs have great potential for macromolecule loading. Arrestin domain-containing protein1-mediated microvesicles, similar to EVs, could selectively recruit targeted proteins, therapeutic RNAs, and the genome-editing CRISPR-Cas9/guide RNA complex 19. Moreover, a cellular nanoporation (CNP) biochip technique was applied to achieve large-scale production and targeted nucleotide sequence encapsulation in exosomes 20. Besides, recent studies revealed that RNA-binding proteins (RBPs) are crucial in sorting RNAs into EVs during biosynthesis 21-23. Therefore, EV membrane proteins, such as CD9 or CD63, were engineered to fuse with RBPs for selective enrichment of target RNAs. A loading approach was proposed, in which MS2 bacteriophage coat protein (MCP) was fused to the EV membrane protein Lamp2b. An increased loading efficiency of targeted RNAs was observed when the cognate MS2 stem-loop was engineered into cargo RNAs 24. In our previous studies, CD9-HuR was used to sort AU-rich element-containing RNAs and Lamp2b-MCP for sorting the MS2 stem-loop-containing RNAs into exosomes 25, 26. However, the mRNAs delivered by this strategy tend to confine within the endosomes due to the high affinity between target mRNAs and the membrane-anchored fused RBPs 26. To solve this problem, we recently engineered EVs by fusing zinc finger (ZF) motifs with CD9 and designed a DNA aptamer to recognize and facilitate targeted mRNA encapsulation into CD9-ZF-engineered EVs. The DNA aptamer binding to targeted mRNA could be degraded by electroporating DNase before delivery to recipient cells, resulting in controlled, targeted mRNA release 27.

We developed the controllable release of therapeutic mRNA to simplify the EV delivery strategy by avoiding electroporation and exogenous DNase application. In this study, we proposed an improved EV-based strategy for enhanced *Ldlr* mRNA loading efficiency in EVs and for facilitated mRNA cargo release into the cytosol of recipient cells. The donor cells were forced to express the CD9-MCP fusion protein and *Ldlr* mRNA simultaneously. With a customized MS2 stem-loop-containing RNA aptamers base-pair matched with *Ldlr* mRNA, target mRNA encapsulation into EVs was enriched via MS2-MCP interaction. In the recipient cells receiving these therapeutic EVs, additional treatment of another type of EVs containing “*Ldlr* releaser”, with a sequence similar to the recognition sites in target mRNA, could release the therapeutic mRNA from CD9-MCP for efficient translation. The proposed strategy was demonstrated to be efficient in loading and delivering functional low-density lipoprotein receptor (*Ldlr*) mRNA with therapeutic potential in the *Ldlr* knockout mouse model.

## Results

### Specific enrichment of *Ldlr* mRNA in CD9-MCP-engineered EVs

**Figure 1:**
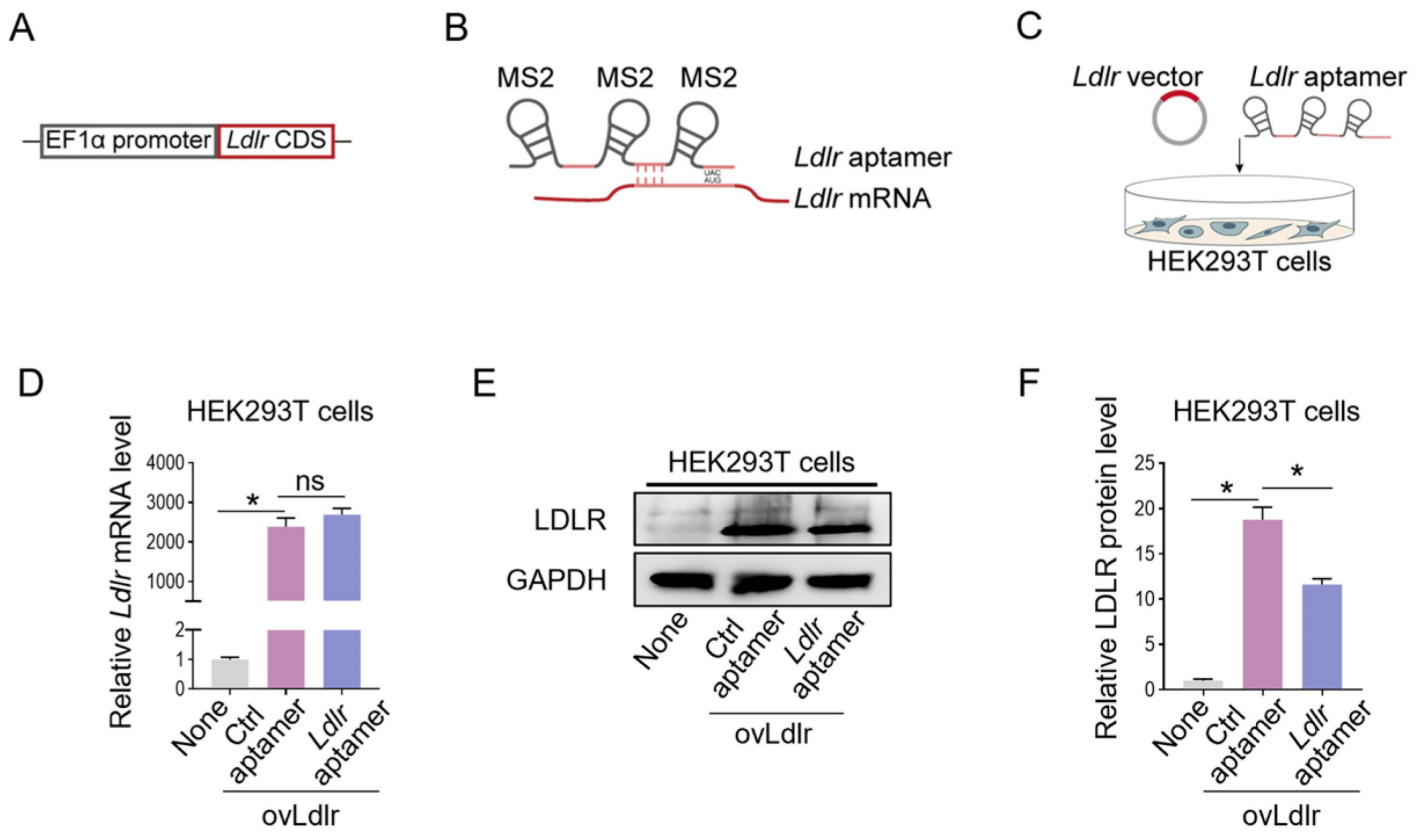
Construction of the *Ldlr* expression vector and aptamer: (A) Schematic illustration of *Ldlr*-expressing vector construction. The CDS of *Ldlr* was cloned downstream of the EF1α promoter. (B) Schematic illustration of the interaction between *Ldlr* mRNA and *Ldlr* aptamer. The *Ldlr* aptamer contains 3 MS2 stem-loop sites and a sequence region base-pair matched with *Ldlr* mRNA near the translation initiation site. (C) Schematic illustration of co-transfection of *Ldlr*-expressing vector (ovLdlr) and *Ldlr* aptamer into HEK293T cells (D) Expression of *Ldlr* mRNA in HEK293T cells treated as indicated. *Gapdh* served as the internal control. (E) Western blot analysis of LDLR protein in HEK293T cells with indicated treatments. GAPDH expression served as the loading control. The data shown are representative of 3 independent experiments. (F) Quantification of Western blot band intensity by densitometry. Data are presented as mean±SEM of 3 independent experiments. *P<0,05 by one-way ANOVA. ns, no significance.

We first generated an *Ldlr*-expressing vector by cloning the *Ldlr* coding sequence (CDS) downstream of the EF1α promoter (Figure 1A). Theoretically, the transcribed *Ldlr* mRNA could interact with a customized aptamer (denoted as *Ldlr* aptamer), which included a sequence base-pair matching with *Ldlr* mRNA near the translation initiation codon and 3 MS2 stem-loop sites. The 19-base RNA stem-loop structured MS2 stem-loop site could be recognized by MCP (Figure 1B, Figure S1A). As the recruitment of ribosomal subunits upstream of the initiation codon is crucial for protein synthesis initiation 28, *Ldlr* mRNA recognition by the *Ldlr* aptamer near the initiation codon might disturb ribosome assembly and prevent translation initiation. We verified this hypothesis by co-transfecting the *Ldlr*-expressing vector and the synthesized *Ldlr* aptamer into HEK293T cells (Figure 1C). Co-transfection with *Ldlr* aptamer did not change the *Ldlr* mRNA expression (Figure 1D), but significantly lowered LDLR protein expression (Figure 1E-F). In contrast, Co-transfection with the control aptamer (denoted as Ctrl aptamer), which did not base-pair with *Ldlr* mRNA, had no such effects. The data suggested that *Ldlr* aptamer could inhibit *Ldlr* mRNA translation.

**Figure 2:**
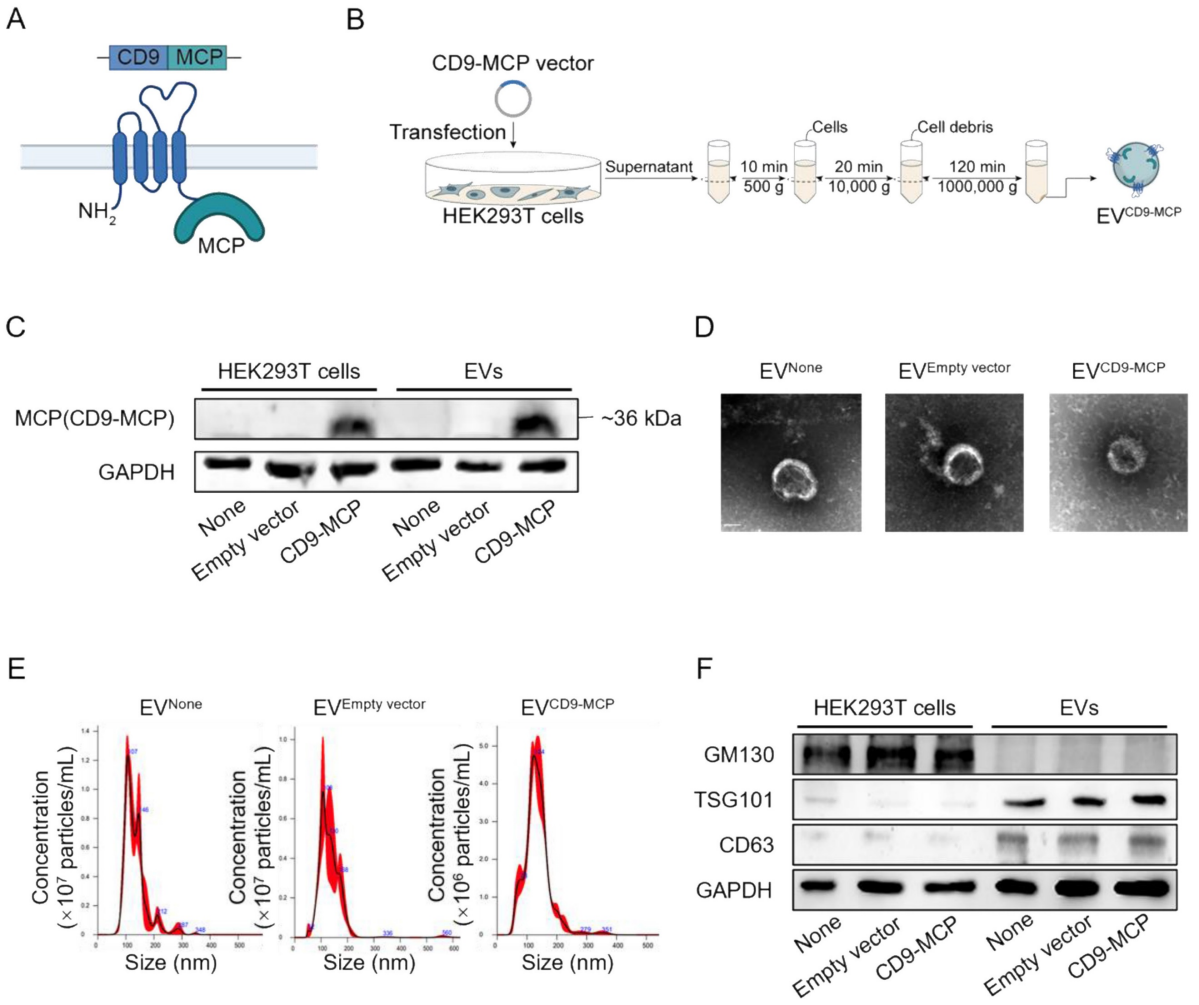
EVs engineered with CD9-MCP fusion protein: (A) Schematic illustration of engineering strategy and membrane localization of the CD9-MCP fusion protein. MCP was designed to fuse to the C-terminus of CD9. (B) Schematic illustrating the preparation and isolation of CD9-MCP-engineered EVs. (C) Representative Western blot analysis of CD9-MCP fusion protein expression in HEK293T cells and derived EVs with indicated treatments. Equal amounts of protein samples were loaded, and GAPDH served as the loading control. (D) Representative TEM images of indicated EVs. (E) Size distribution of indicated EVs. (F) Western blot analysis of the inclusive EVs markers TSG101, CD63, and the exclusive marker GM130.

In subsequent experiments, we explored whether the *Ldlr* aptamer could promote *Ldlr* mRNA encapsulation into EVs engineered with CD9-MCP, in which MCP was fused to the C-terminus of CD9 (Figure 2A). HEK293T cells without any treatment (None), transfected with empty vector, or CD9-MCP recombinant plasmid were used, and the EVs isolated by ultracentrifugation were denoted as EV^None^, EV^Empty vector^, and EV^CD9-MCP^ respectively. The CD9-MCP fusion protein was highly expressed in HEK293T cells transfected with the CD9-MCP recombinant plasmid compared with the None or Empty vector groups (Figure 2B-C). Also, compared with EV^None^ or EV^Empty vector^, EVs derived from HEK293T cells transfected with EV^CD9-MCP^ had abundant CD9-MCP fusion protein (Figure 2C). No noticeable change in EV^CD9-MCP^ size distribution and morphology was observed, as characterized by nanoparticle tracking analysis and transmission electron microscopy (Figure 2D-E). The average diameters of isolated vesicles were 132.0±2.3 (EV^None^), 133.6±3.7 (EV^Empty vector^), 134.1±2.6 (EV^CD9-MCP^), and no significant differences were detected among the 3 groups. Western blot analysis of the exosomal inclusive markers CD63, TSG101, and the exclusive marker GM130 further verified that most engineered EVs were exosomes (Figure 2F).

**Figure 3:**
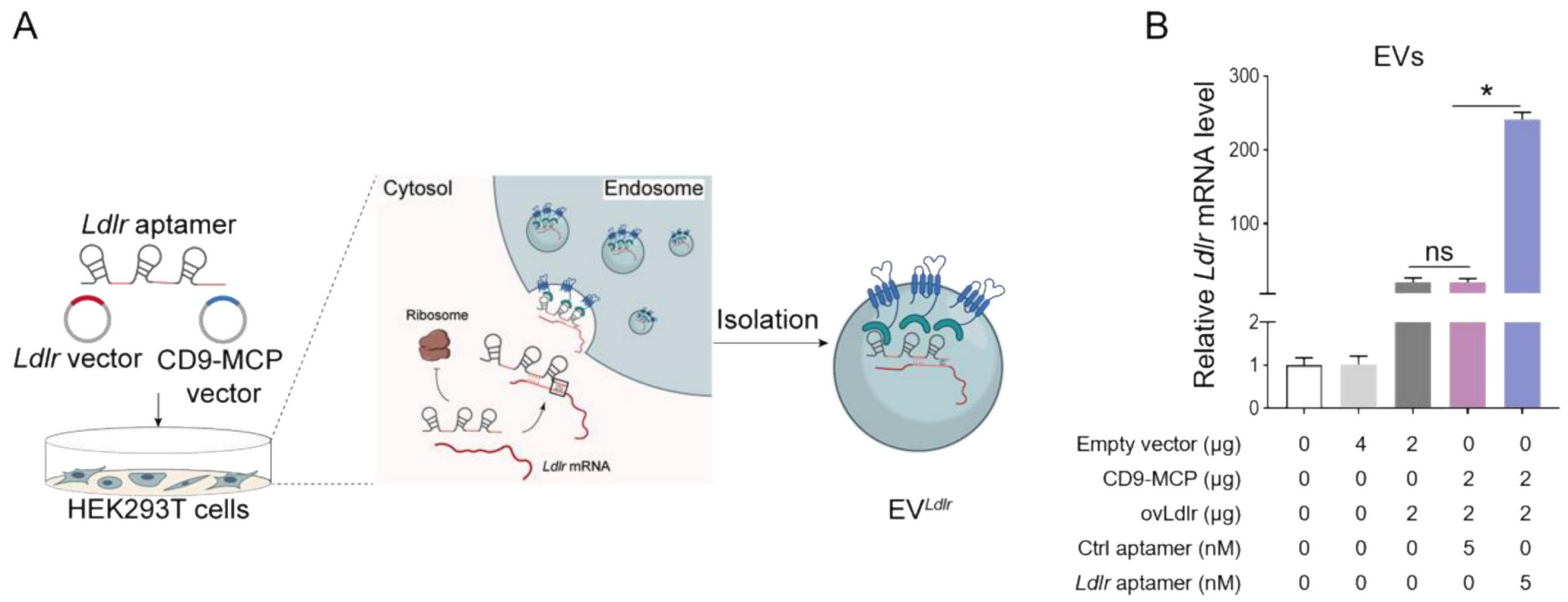
*Ldlr* aptamer promotes *Ldlr* mRNA sorting into CD9-MCP-engineered EVs: (A) Schematic illustration of *Ldlr* mRNA enriched in CD9-MCP-engineered EVs. *Ldlr*-expressing vector, *Ldlr* aptamer, and CD9-MCP vector were simultaneously transfected into HEK293T cells. *Ldlr* mRNA was expressed by the *Ldlr*-expressing vector and recognized by the *Ldlr* aptamer, preventing translation. The *Ldlr* aptamer promoted *Ldlr* mRNA enrichment in CD9-MCP-engineered EVs through MS2/MCP binding. (B) qPCR analysis of *Ldlr* mRNA in EVs derived from cells treated as indicated. Data are presented as mean±SEM of 3 independent experiments. *P<0.05 one-way ANOVA.

*Ldlr*-expressing vector, CD9-MCP recombinant vector, and *Ldlr* aptamer were simultaneously transfected into HEK293T cells, MS2 stem-loop sites in *Ldlr* aptamer were expected to facilitate *Ldlr* mRNA sorting into CD9-MCP-engineered EVs via MS2-MCP interaction (Figure 3A). The efficiency of *Ldlr* aptamer for sorting* Ldlr* mRNA into EVs was confirmed by qPCR analysis of *Ldlr* mRNA abundance in EVs. Compared with the controls, transcribed *Ldlr* mRNA was selectively encapsulated into the CD9-MCP-engineered EVs, while the *Ldlr* aptamer further promoted* Ldlr* mRNA encapsulation (Figure 3B). Moreover, with the enrichment of the *Ldlr* mRNA in EV*^Ldlr^*, there was no space for other mRNAs in the EVs. In other words, EVs had much fewer nonspecific mRNAs, which could be seen from the higher Ct value of* Gapdh* per EV (Figure S2).

The advantage of the proposed strategy for loading efficiency was further confirmed by including an *Ldlr*-MS2-expressing vector as another control in which the transcribed *Ldlr* mRNA was flanked with 3 MS2 stem-loop sites in the 3' UTR region (Figure S3A) and could be encapsulated into CD9-MCP-engineered EVs via MS2-MCP interaction (Figure S3B). When the *Ldlr* mRNA levels were comparable between *Ldlr*-MS2-transfected and *Ldlr*+*Ldlr* aptamer-transfected HEK293 cells (Figure S3C), LDLR protein expression in the *Ldlr*-MS2 group was much higher than in the *Ldlr*+*Ldlr* aptamer group (Figure S3D-E), further confirming that *Ldlr* aptamer could repress *Ldlr* mRNA translation. Thus, due to the mRNA translation inhibition, *Ldlr* mRNA abundance was higher in the EVs in the *Ldlr*+*Ldlr* aptamer group than in the *Ldlr*-MS2 group (Figure S3F). It is plausible that due to its small size, translationally repressed *Ldlr* mRNA was easily recruited into CD9-MCP-engineered EVs.

### EV-mediated functional *Ldlr* mRNA delivery *in vitro*

*Ldlr* mRNA release to the cytosol is a prerequisite for its translation. To this end, an *Ldlr* releaser with a similar sequence as the *Ldlr* AUG region and based-paired with the *Ldlr* aptamer was synthesized. The higher affinity of the aptamer to *Ldlr* releaser than to *Ldlr* mRNA was achieved by base-paring (Figure S1), allowing the *Ldlr* releaser to replace *Ldlr* mRNA with high efficiency. *Ldlr* releaser or Ctrl releaser was transfected into HEK293T cells, and the EVs (denoted as EV*^Ldlr^*^ releaser^ or EV^Ctrl releaser^) were collected (Figure S4A). Nanoparticle tracking analysis and transmission electron microscopy showed that *Ldlr* releaser or Ctrl releaser encapsulation did not change the size and morphology of engineered EVs compared with non-loading EVs (Figure S4B-C). Western blot analysis revealed a similar expression pattern of the exosomal inclusive markers CD9 and TSG101 and exclusive marker GM130, validating the exosomal characteristics of the EVs (Figure S4D). qPCR analysis confirmed that *Ldlr* releaser or Ctrl releaser was successfully transfected into the recipient cells and loaded into EVs compared with U6 snRNA (Figure S4E-F). Theoretically, a 10-fold excess of *Ldlr* releaser is needed to release *Ldlr* mRNA competitively. Notably, the copy number of *Ldlr* releaser per EV was more than 60-fold higher than Ldlr mRNA per EV, indicating EV*^ Ldlr^*^releaser^/EV*^Ldlr^* at the ratio of 1:10 translated into the *Ldlr* releaser/*Ldlr* mRNA copy number ratio of 6:1.

**Figure 4:**
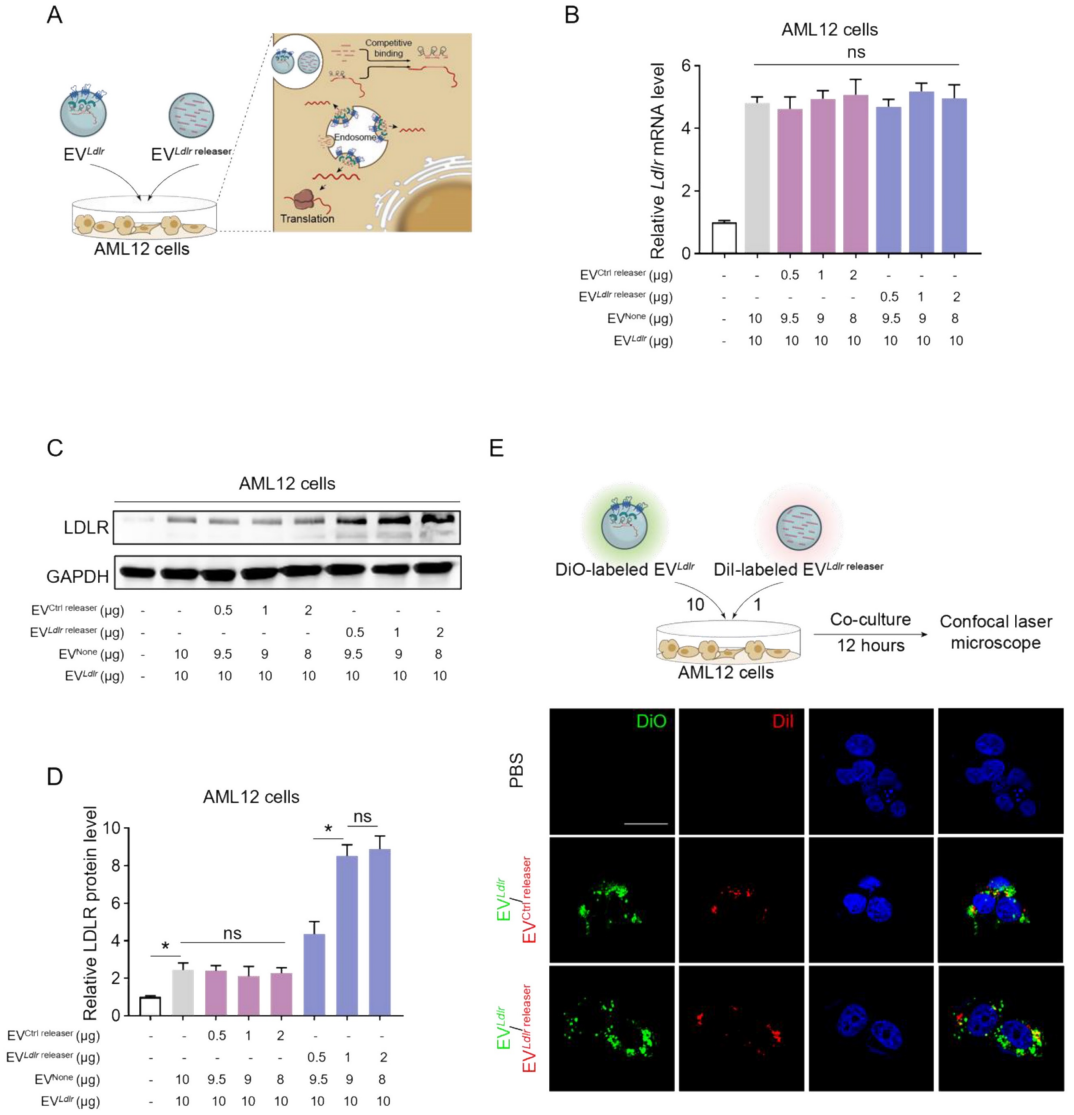
EV*^Ldlr^*/EV*^Ldlr^* ^releaser^ efficiently delivers *Ldlr* mRNA for translation *in vitro*: (A) Schematic illustration of the EV-mediated *Ldlr* mRNA and *Ldlr* releaser delivery and mRNA release for efficient translation (B) qPCR analysis of* Ldlr* mRNA in AML12 cells. EV*^Ldlr^*^ releaser^ or EV^Ctrl release^, together with EV*^Ldlr^* at the indicated amounts, were incubated with AML12 cells. EV^None^ was applied to ensure an equal amount of EVs in each group. Data are presented as mean±SEM of 3 independent experiments. ns, no significance. (C) Western blot analysis of LDLR protein expression in AML12 cells treated as indicated. The data shown are representative of 3 independent experiments. (D) Quantification of Western blot bands by densitometry. The data are presented as mean±SEM of 3 independent experiments. *P<0.05 by one-way ANOVA. ns, no significance. (E) Representative fluorescence microscopy images showing endocytosis of EVs by AML12 cells. DiO-labeled EV*^Ldlr^* and DiI-labeled EV*^Ldlr^*^ releaser^ (or EV^Ctrl releaser^) were incubated with the cells and visualized by fluorescence microscopy. Nuclei were counterstained by Hoechst. Treatment with phosphate-buffered saline (PBS) served as the negative control. Scale bar=20 μm.

We incubated AML12 cells with EV^Ctrl releaser^ and EV*^Ldlr^* at the ratio of 1:20, 1:10, and 1:5. EV^None^, the EV derived from HEK293T cells without any treatment, was included to ensure equal amounts of EVs in each group. The *Ldlr* mRNA level increased in AML12 cells after receiving EV*^Ldlr^*^ releaser^/EV*^Ldlr^* treatment (Figure 4B). Western blot analysis showed significantly increased LDLR protein expression in the EV*^Ldlr^*^releaser^/EV*^Ldlr^* treatment group compared with EV^Ctrl releaser^/EV*^Ldlr^* treatment. Moreover, EV*^Ldlr^*^releaser^/EV*^Ldlr^* at the 1:10 ratio increased LDLR expression to nearly 6-fold, while higher EV*^Ldlr^*^releaser^ had no additional effects (Figure 4C, D). Especially, the fold-change could not be achieved by increasing the EV amount, as the cell endocytosis ability was limited (Figure S5A-B). EV tracking analysis confirmed that DiO-labeled EV*^Ldlr^* and DiI-labeled EV*^Ldlr^*^ releaser^ (or EV^Ctrl releaser^) could be endocytosed by AML12 cells efficiently, ensuring the competitive binding (Figure 4E).

We verified whether EV*^ Ldlr^* ^releaser^/EV*^Ldlr^* treatment at the ratio of 1:10 completely released the mRNA by transfecting AML12 cells with different doses of *Ldlr* releaser, followed by incubation with 10 µg EV*^Ldlr^* (Figure S6A). The LDLR protein expression was enhanced by increasing the dose of the *Ldlr* releaser, However, transfection at 0.5 nM or 2.5 nM *Ldlr* releaser or 1 µg EV^releaser^ had similar releasing effects, suggesting that transfection at 0.5 nM or 1 µg EV*^ Ldlr^* ^releaser^ was enough to release all the *Ldlr* mRNA delivered by 10 µg EV*^Ldlr^* (Figure S6B). Notably, liposome-based delivery needed more *Ldlr* releaser to completely release *Ldlr* mRNA than EV*^Ldlr^*^ releaser^, which might be attributed to high endocytosis efficiency and easy endosome escape for EV-based delivery. It is also important to note that EV*^Ldlr^*^releaser^/EV*^Ldlr^*-mediated *Ldlr* mRNA delivery and subsequent translation were much higher than the same amount of EV-based *Ldlr*-MS2 delivery (Figure S6C). Collectively, these results showed that EV*^Ldlr^*, together with EV*^Ldlr^*^releaser^ at the ratio of 10:1, should be an efficient platform for delivering functional *Ldlr* mRNA into recipient cells.

### Efficient *in vivo* delivery of Ldlr mRNA to the liver by EV*^Ldlr^*/EV*^Ldlr^*^releaser^

**Figure 5:**
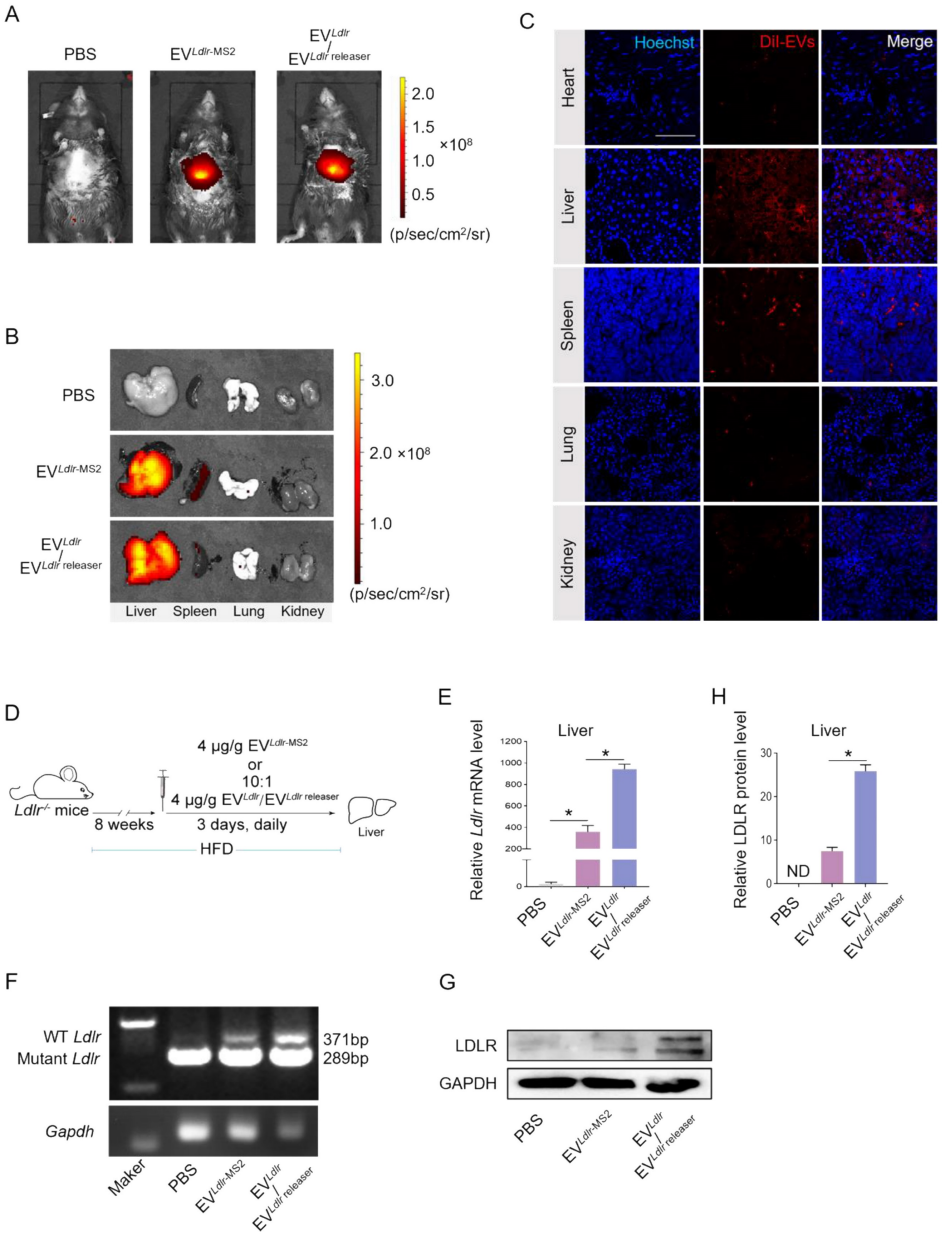
EV*^Ldlr^*/EV*^Ldlr^*^ releaser^ effectively delivers translationally accessible *Ldlr* mRNA *in vivo*: (A) Representative IVIS images showing the distribution of EVs. Mice were injected with PBS, 100 μg DiR-labeled EV*^Ldlr^*^-MS2^ or EV*^Ldlr^*/EV*^Ldlr^*^releaser^ (10:1) in 100 μl via the tail vein, and IVIS imaging was performed 4 h after injection. (B) The distribution of DiR-labeled EVs in different organs as visualized by IVIS (C) Representative fluorescence microscopic images of the localization of DiI-labeled EVs in indicated organs. Scale bar = 100 μm (D) Schematic illustration of the experimental procedure. *Ldlr*^-/-^ mice 6-8 weeks of age were fed with HFD for 8 weeks, followed by the tail vein injection of EVs daily for 3 days. (E) qPCR analysis of exogenous *Ldlr* mRNA in livers of mice treated as indicated. (F) Representative semi-quantitative PCR analysis of* Ldlr* mRNA expression in livers from mice after indicated treatments. The 371 bp band represents the wild-type *Ldlr* from EVs, while the 289 bp band represents endogenous mutated *Ldlr* in *Ldlr*^-/-^ mice. (G) Western blot analysis of LDLR expression in livers from mice treated as indicated. Representative data from 3 independent experiments. (H) Quantification of Western blot bands by densitometry. Data are presented as mean±SEM. *P<0.05 by one-way ANOVA.

*In vivo* distribution in target organs is a prerequisite for the therapeutic effects of any proposed strategy. After tail vein injection, we profiled the in vivo distribution of EV*^Ldlr^*^-MS2^ or EV*^Ldlr^*/EV*^Ldlr^*^ releaser^. DiR-labeled EVs were tracked and visualized by the *in vivo* imaging system (IVIS) and found that EV*^Ldlr^*^-MS2^ or EV*^Ldlr^*/EV*^Ldlr^*^ releaser^ had a similar distribution profile, mainly in the liver and spleen (Figure 5A-B). In addition, DiI-labeled EVs were tracked and visualized by fluorescence microscopy in tissue sections, confirming the same *in vivo* distribution profile between EV*^Ldlr^*^-MS2^ and EV*^Ldlr^* /EV*^Ldlr^*^ releaser^ treatment (Figure 5C). The strong liver tropism might be ascribed to two aspects. On the one hand, Kuppfer cells in the liver are one of the key recipient cell types of the EVs due to their high phagocytosis capacity, and on the other hand, hepatocytes are also the recipient cells due to the sinusoidal structure in the liver and their high uptake ability 29-31. The strong tropism in the spleen can be attributed to the presence of abundant macrophages. Collectively, the data suggested that EV could efficiently deliver mRNA into hepatocytes.

The therapeutic effects of EV*^Ldlr^*^-MS2^ or EV*^Ldlr^* /EV*^Ldlr^*^ releaser^ following 8 weeks of a high-fat diet were explored in *Ldlr*^-/-^ mice receiving PBS, EV*^Ldlr^*^-MS2^, or EV*^Ldlr^* /EV*^Ldlr^*^ releaser^ (at the ratio of 10:1) (Figure 5D). Consistent with the higher abundance of *Ldlr* mRNA in EV*^Ldlr^*/EV*^Ldlr^*^ releaser^ than in EV*^Ldlr^*^-MS2^, EV*^Ldlr^* /EV*^Ldlr^*^ releaser^ treatment increased the wild-type *Ldlr* mRNA in the liver to a greater extent than EV*^Ldlr^*^-MS2^ treatment, as detected by nested PCR and semi-quantitative PCR (Figure 5E-F, and Figure S7A-B). Accordingly, LDLR protein expression in the liver was also significantly higher, with a nearly 3-fold increase after EV*^Ldlr^*/EV*^Ldlr^*^ releaser^ treatment than EV*^Ldlr^*^-MS2^ treatment (Figure 5G-H). These results revealed that the proposed EV*^Ldlr^*/EV*^Ldlr^*^ releaser^ strategy could deliver *Ldlr* mRNA into the liver *in vivo* and release* Ldlr* mRNA into the cytosol for efficient translation.

### Amelioration of liver steatosis and atherosclerosis by EV*^Ldlr^*/EV*^Ldlr^*^releaser^ treatment

**Figure 6:**
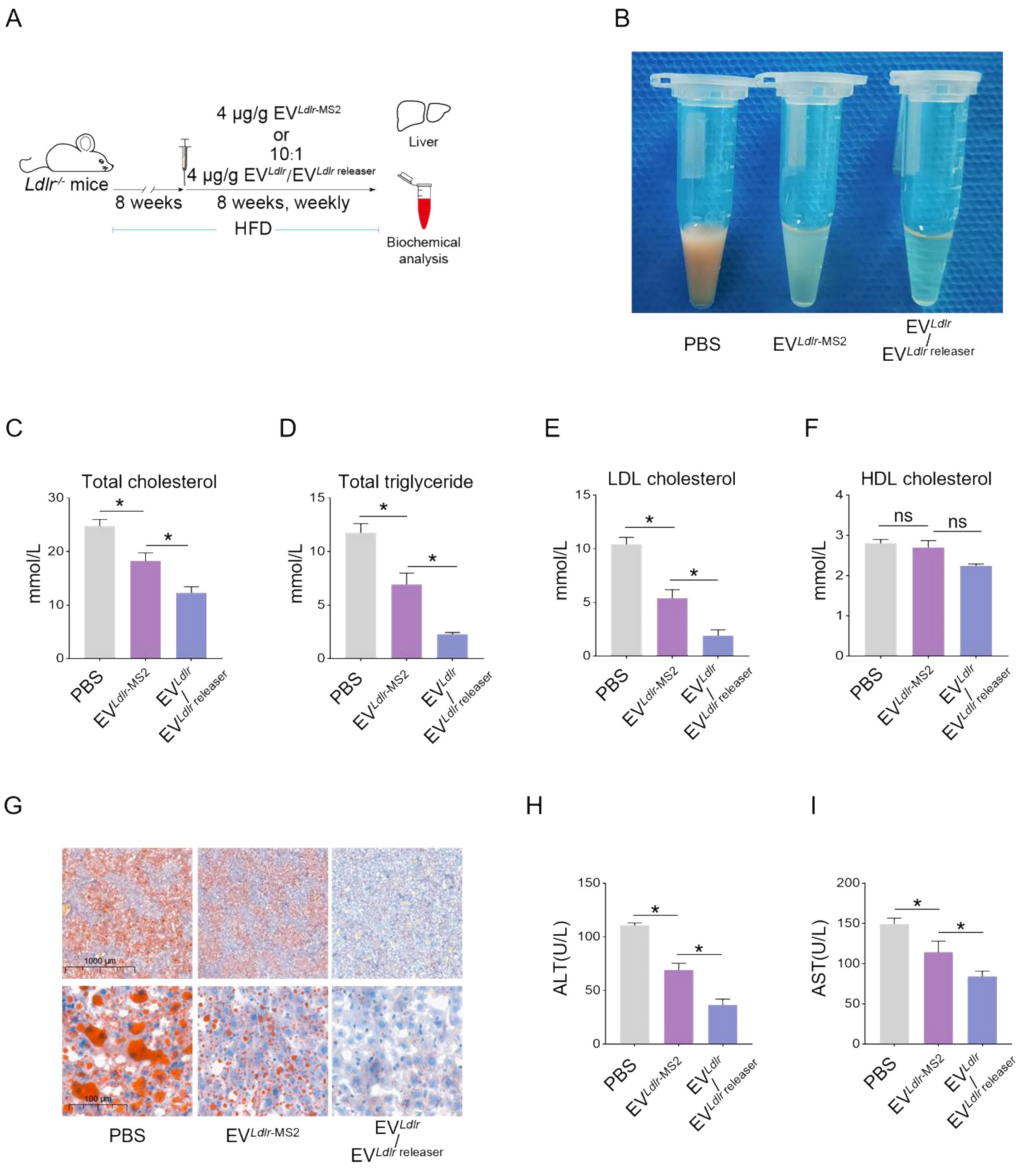
EV*^Ldlr^*/EV*^Ldlr^*^ releaser^ treatment ameliorates abnormal lipid metabolism in *Ldlr*^-/-^ mice: (A) Schematic illustration of the experimental procedure. *Ldlr*^-/-^ mice aged 6-8 weeks were first fed with HFD for 8 weeks, followed by the tail vein injection of PBS or indicated EVs weekly for 8 weeks as indicated. At the end of the experiments, mice were sacrificed for systemic analysis. (B) Representative images of serum appearance from mice treated as indicated. Analysis of (C) total cholesterol, (D) total triglyceride, (E) LDL-C, and (F) HDL-C in *Ldlr*^-/-^ mice treated as indicated. (G) Representative images of Oil Red O staining of the liver in *Ldlr*^-/-^ mice treated as indicated. Analysis of (H) plasma ALT and (I) AST. Data are presented as mean±SEM. n=6. *P<0.05 by one-way ANOVA. ns, no significant difference.

**Figure 7:**
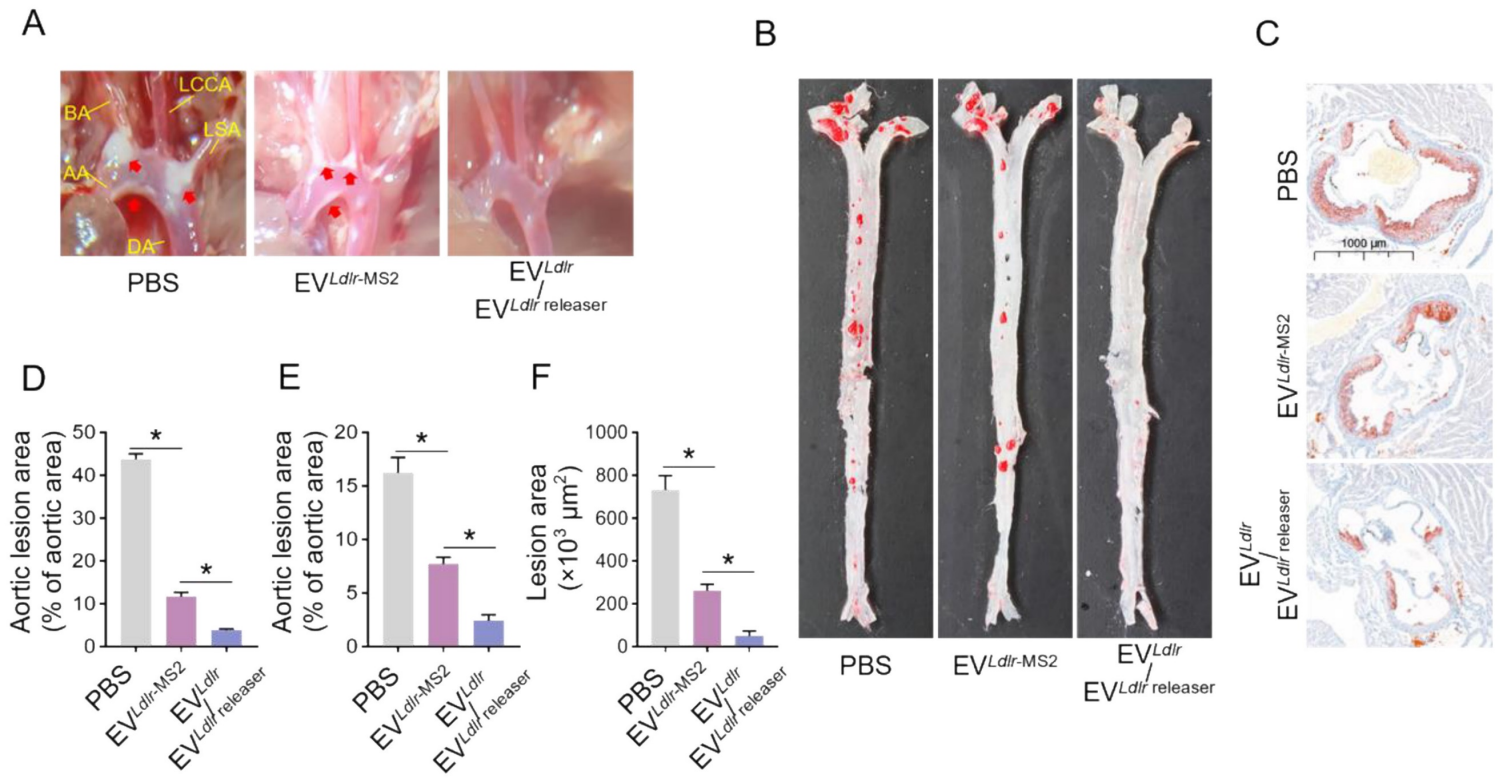
EV*^Ldlr^*/EV*^Ldlr^*^ releaser^ treatment alleviates atherosclerotic lesions in *Ldlr*^-/-^ mice: (A) Representative images of the atherosclerosis lesions at the aortic arch in mice with indicated treatments. *Ldlr*^-/-^ mice aged 6-8 weeks were fed with HFD for 8 weeks, followed by the tail vein injection of PBS or indicated EV combinations weekly for 8 weeks as indicated. AA, ascending aorta; BA, brachiocephalic artery; LCCA, left common carotid artery; LSA, left subclavian artery; DA, descending aorta (B) Representative images of Oil Red O staining of the atherogenic lesion areas in mice treated as above (C) Representative images of the cross-sectional view of aortic roots stained with Oil Red O in mice treated as above (D) Percentage of the atherosclerotic area in the aortic arch in mice treated as above (E) Percentage of the atherosclerotic region in B. (F) Statistical analysis of the Oil O red positive plaque area in C. Data are presented as mean ± SEM. n=6. *P<0.05 by one-way ANOVA.

Subsequently, we evaluated the therapeutic effects of the EV*^Ldlr^*/EV*^Ldlr^*^ releaser^ in *Ldlr*^-/-^ mice on a high-fat diet treated with indicated EVs weekly for 8 weeks (Figure 6A). Treatments with PBS or EV*^Ldlr^*^-MS2^ were included as controls. The serum was extremely milky in the PBS group, semi-transparent in the EV*^Ldlr^*^-MS2^ group, and nearly transparent in EV*^Ldlr^*/EV*^Ldlr^*^ releaser^ group (Figure 6B). Besides, both EV*^Ldlr^*^-MS2^ and EV*^Ldlr^*/EV*^Ldlr^*^ releaser^ treatments decreased the total cholesterol, triglyceride, and LDL-C levels, while the fold-change induced by EV*^Ldlr^*/EV*^Ldlr^*^ releaser^ was much larger (Figure 6C-E). However, no significant difference in the HDL-C level was observed among the 3 groups (Figure 6F). Oil Red O staining of liver sections consistently showed reduced lipid accumulation in hepatocytes following EV*^Ldlr^*/EV*^Ldlr^*^ releaser^ treatment (Figure 6G). Accordingly, plasma AST and ALT decreased after EV*^Ldlr^*/EV*^Ldlr^*^ releaser^ treatment (Figure 6H-I). EV*^Ldlr^*^-MS2^ treatment also reduced lipid accumulation in hepatocytes and decreased plasma AST and ALT, though the effects were not as prominent as with the EV*^Ldlr^*/EV*^Ldlr^*^ releaser^ treatment (Figure 6G-I). Consistent with the rescued lipid metabolism, EV treatment, especially EV*^Ldlr^*/EV*^Ldlr^*^ releaser^ treatment, decreased the number and size of atherosclerosis plaques, as detected by the gross view of the blood vessels, Oil O Red staining, and the quantification data (Figure 7A-F). Especially, all groups had no significant differences in body weights after treatment (Figure S8). In summary, the proposed EV*^Ldlr^*/EV*^Ldlr^*^ releaser^ delivery strategy efficiently restored the gene expression at the protein level in the liver and ameliorated the disease phenotype, including steatosis, high LDL-C, and atherosclerosis.

## Discussion

In the present study, we have developed an EV-based mRNA delivery strategy in which the therapeutic mRNA was selectively enriched in EVs by a customized RNA aptamer in donor cells and efficiently released by a releaser RNA sequence for translation in recipient cells. We efficiently delivered *Ldlr* mRNA using the proposed strategy to the liver cells, restoring LDLR expression and ameliorating the phenotype of high LDL cholesterol, atherosclerosis, and steatosis in the *Ldlr*^-/-^ mouse model.

EV-mediated delivery of therapeutic mRNAs has shown great potential for clinical translation. However, the low loading efficiency, especially for target mRNAs of large size, precludes its wide application 32, 33. Recent studies revealed that RNA cargos with specific sequences could be recognized by corresponding RNA-binding proteins, allowing selective encapsulation of RNA into EVs with high efficiency. Exosomal proteins fused with specific RBPs have been developed to enrich RNAs of interest 24, 34, 35. In this study, a customized RNA aptamer could be recognized by the RNA-binding protein MCP via the MS2 stem-loop and interacted with *Ldlr* mRNA by complementary base pairing with the sequence around the translation initiation region. The RNA aptamer selectively recruited the mRNA to the EVs and also repressed *Ldlr* mRNA translation, possibly by disturbing translation initiation and ribosome movement, allowing much easier mRNA encapsulation into EVs. This “indirect” strategy has the advantage of designing the RNA aptamer for any mRNA of interest without manipulating the therapeutic mRNA itself. Although a tailored RNA aptamer with MS2 was created in this study, its optimization to increase* Ldlr* mRNA enrichment is worth further exploration, especially for the recognition region corresponding to the AUG region, the length of the sequence, and the specificity to the target mRNA.

mRNA cargos must be released to the cytosol to serve as translation templates. EV cargos are released when the EVs fuse with the plasma membrane or endosome membrane. Thus, target mRNAs delivered by the proposed EV strategy are restricted due to the MCP-aptamer-RNA interaction, making the target mRNA inaccessible for translation 36-38. We designed the *Ldlr* releaser for the efficient release of the *Ldlr* mRNA with two specific features: 1) *Ldlr* releaser was endowed with a superior binding ability to the aptamer as the matched nucleotides to *Ldlr* aptamer were longer than that of *Ldlr* mRNA, and 2) *Ldlr* releaser was encapsulated in EVs and simultaneously delivered to recipient cells with EV*^Ldlr^*. As a small RNA, a large amount of releaser could be encapsulated per EV, and therefore, much less EV*^Ldlr^*^ releaser^ was needed to release the mRNA. We found that EV*^Ldlr^*^ releaser^/EV*^Ldlr^* at the ratio of 1:10 was sufficient to release the *Ldlr* mRNA, producing a more than 3-fold increase in protein expression. Notably, in the titration experiment, liposome-based delivery required more *Ldlr* releaser to release *Ldlr* mRNA than EV*^Ldlr^*^ releaser^ completely. This could be explained by the possibility that EV might have high endocytosis efficiency and easier endosome escape.

The flexible strategy in designing the RNA aptamer and releaser might endow the aptamers with additional functions. For example, it has been shown that inhibition of proprotein convertase subtilisin/kexin type 9 (PCSK9) by small interfering RNA (siRNA) could lower LDL cholesterol in FH 16, 39. Theoretically, endowing the aptamers or releasers with the PCSK9 knockdown feature would be beneficial for improving the lipid profile.

## Conclusion

We have developed an improved strategy for efficient encapsulation of* Ldlr* mRNA into EVs in donor cells with a controllable release of the therapeutics in recipient cells. In the donor cells, CD9-MCP fusion protein, *Ldlr* mRNA, and a customized MS2 stem-loop-containing RNA aptamer sorted the *Ldlr* mRNA into EVs. In the recipient cells, EV-mediated delivery of *Ldlr* releaser competitively interacted with the aptamer and released the *Ldlr* mRNA for efficient translation. The proposed strategy demonstrated improved therapeutic effects in FH treatment and holds great potential for delivering other therapeutic mRNAs for a broad range of diseases.

## Materials and Methods

### Design and synthesis of plasmid construction and aptamers

Plasmids expressing CD9-MCP, *Ldlr,* and *Ldlr*-MS2 were synthesized by Genscript Biotech Corporation. *Ldlr* aptamer, Ctrl *Ldlr* aptamer, *Ldlr* releaser, and Ctrl releaser were also synthesized by Genscript Biotech Corporation. The detailed sequences are listed in Table S1-2.

### Cell culture and transfection

HEK293T cells and mouse liver 12 (AML12) cells were cultured in complete media containing high glucose Dulbecco's Modified Eagle Medium (DMEM) (Logan, Utah, USA) with 1% penicillin-streptomycin and 10% fetal bovine serum (FBS) (Logan, Utah, USA). Cells were cultured in a humidified incubator with 5% CO2 at 37°C. Fresh medium was replaced every two days. For *in vitro* treatment of EVs in cell culture, 10 μg EVs per well were cocultured with HEK293T cells in 6-well plates. Lipofectamine 2000 (Invitrogen, Carlsbad, CA, USA) was used to transfect plasmids or designed aptamers as instructed by the manufacturer's protocol. Before transfection, cells were pretreated with serum-free DMEM for 6 h.

### EV isolation and characterization

EVs were collected by ultracentrifugation as described previously with minor modifications 40. Cells were cultured in an FBS-free medium. The culture medium was centrifuged under 500 g for 10 min and then at 10000 g for 20 min to remove cells and debris. The supernatant was then ultracentrifuged at 100000 g for 120 min. The EV pellets were washed once with PBS and were resuspended in PBS or stored at -80 ℃ for subsequent experiments. The EVs were imaged by transmission electron microscopy (JEM-200EX TEM, Tokyo, Japan) and diluted for size distribution analysis by Nanosight. The exosomal marker expression in EVs was analyzed by Western blotting using antibodies against CD9 (ab236630, Abcam), GM130 (sc71166, Santa Cruz), TSG101 (ab83, Abcam), and CD63 (ab217345, Abcam).

### EV labeling and tracing

EVs were labeled by DiO, DiI, or DiR (Beyotime, China) via direct incubation for 30 min, and the free dye was removed by another round of EV isolation. Cells were incubated with DiI- or DiO-labeled EVs (20 μg/mL) in 35 mm confocal dishes for 12 h. Subsequently, the cells were washed with PBS and fixed with 4% paraformaldehyde for 10 min. The nuclei were counterstained with Hoechst for 5 min, and the images were obtained using a confocal microscope. For in vivo tracing of EVs, purified DiR-labeled EVs were injected via the tail vein, and the distribution of EVs was analyzed by an *in vivo* imaging system (IVIS) 4 h later.

### qRT-PCR

Total RNA was extracted using the TRIzol reagent (Invitrogen, USA). Small RNAs (including the aptamer and releaser) and mRNA were reversely transcribed using the miRcute Plus miRNA Synthesis Kit and Transcriptor First-strand cDNA Synthesis Kit (Roche), respectively, following the manufacturers' instructions. qPCR was performed by FastStart Essential DNA Green Master. U6 was used as an internal control, and RNA aptamers and GAPDH (*Gapdh*) were used for the normalization of mRNA abundance. Relative expression was calculated by the 2-ddCt method unless otherwise indicated. The sequences of PCR primers are provided in Table S3.

### Western blots

EVs were collected from the cell culture medium by ultracentrifugation. The protein concentration in each sample was measured by the Pierce BCA Protein Assay Kit (23225, Thermo Scientific™). About 40 μg EV protein samples or cell/tissue lysates were separated by SDS-PAGE and transferred to the nitrocellulose filter membrane, blocked with 3% nonfat milk in TBST for 1 h, and incubated with primary antibodies: anti-GAPDH polyclonal antibody (D110016-0100, BBI Life Sciences), anti-GM130 (11308-1-AP), anti-CD9 (ab92726, Abcam), anti-TSG101 (ab83, Abcam), anti-MCP (ABE76, Merck Millipore), and anti-LDLR (ab52818, Abcam) overnight at 4°C. After washing in TBST, the membranes were incubated with secondary antibodies for 1 h at room temperature, and the chemiluminescence reagent (Millipore, Billerica, MA, USA) was applied to visualize the blots. The densities of the immunoreactive bands were analyzed by Image Lab software (Bio-Rad Laboratories, Hercules, CA, USA).

### Animal experiments

All animal experiments were approved by the Animal Care and Use Committee of Air Force Medical University. *Ldlr*^-/-^ mice were purchased from the Model Animal Research Center of Nanjing University and were raised on a high-fat diet for 8 weeks. To analyze the EV-based delivery efficiency, 4 μg/g EVs were injected via the tail vein once a day for 3 days. Mice were sacrificed at the end of the experiment, and livers were harvested for qPCR and Western blotting. For the therapeutic efficacy evaluation, *Ldlr*^-/-^ mice were fed a high-fat diet for 8 weeks before the treatment. PBS or indicated EVs at the dosage of 4 μg/g were injected via the tail vein weekly for 8 weeks. The blood, liver, and aorta were obtained for analysis.

### Histology

The mice were anesthetized with pentobarbital (65 mg/kg, i.p.). After perfusion with PBS, the main organs, including the heart, lung, liver, spleen, and kidney, were dissected. The aorta and heart were exposed, and surrounding tissues were excised. The aorta-to-iliac bifurcation was dissected for Oil-red-O staining. The aortic arch bifurcation view was photographed by a digital camera under the stereomicroscope. The harvested main organs/tissues were fixed in 4% paraformaldehyde for 1 h and then embedded in OCT compound. Embedded tissues were then cut into 5 μm slices for Oil-red-O or H&E staining. Image J was used to analyze the percentage of lesion area. The lesion size and lipid core area were compared.

### Serum biochemistry

Blood samples of mice were obtained after fasting for 8 h. The whole blood samples were kept at room temperature for 2 h or overnight at 4 °C, then centrifuged at 4°C, 4000 g for 15 min. The supernatants were stored at -20°C. The concentration of plasma triglycerides, total cholesterol, HDL cholesterol, LDL cholesterol, ALT, and AST were measured by Chemray 800 at Wuhan Service Biotechnology CO., LTD.

### Statistical Analysis

Quantitative data are presented as mean ± SEM. The Shapiro-Wilk test was used to verify distribution normality. One-way ANOVA with Tukey's posthoc test was used for multiple comparisons among 3 or more groups. Student's t-test or Mann-Whitney U test were used for two-group comparisons for normally or abnormally distributed data. These analyses were performed by GraphPad Prism 8.0 (GraphPad Software, San Diego, CA). *P* < 0.05 indicated statistical significance.

## Supplementary Material

Table S1: Sequence of aptamers and releasers. Table S2: Sequences of primers. Table. S3: Sequences of plasmids. Figure S1: Design of *Ldlr* aptamer and *Ldlr* releaser. Figure S2: qPCR analysis of Ct value of GAPDH in EVs. Figure S3: Construction of EV*^Ldlr^*^-MS2^ and EV loading efficiency of *Ldlr* mRNA. Figure S4. Construction and characterization of EV*^Ldlr^*^ releaser^. Figure S5: LDLR expression in AML12 cells co-cultured with increasing amounts of EV*^Ldlr^*^-MS2^. Figure S6: Competitive binding of *Ldlr* releaser with *Ldlr* aptamer. Figure S7: Illustration of *Ldlr* gene deletion strategy and primer design. Figure S8: Body weights of mice with different treatments.
