# Supplementary material for: Improved extracellular vesicle-based mRNA delivery for familial hypercholesterolemia treatment
Source: Theranostics. 2023 Jun 12;13(10):3467–79. doi: 10.7150/thno.82873 (PMC10283059; doi:10.7150/thno.82873)
Supplement: Supplementary file 1 — Table S1: Sequence of aptamers and releasers. Table S2: Sequences of primers. Table. S3: Sequences of plasmids. Figure S1: Design of Ldlr aptamer and Ldlr releaser. Figure S2: qPCR analysis of Ct value of GAPDH in EVs. Figure S3: Construction of EVLdlr-MS2 and EV loading efficiency of Ldlr mRNA. Figure S4. Construction and characterization of EVLdlr releaser. Figure S5: LDLR expression in AML12 cells co-cultured with increasing amounts of EVLdlr-MS2. Figure S6: Competitive binding of Ldlr releaser with Ldlr aptamer. Figure S7: Illustration of Ldlr gene deletion strategy and primer design. Figure S8: Body weights of mice with different treatments. [file thnov13p3467s1.pdf]

Supplementary Data

Improved extracellular vesicle-based mRNA delivery strategy for familial hypercholesterolemia treatment

Zheng Yang[a,b,c,1], Panpan Ji[d,1], Zhelong Li[e,1], Rongxin Zhang[c,f], Mengying Wei[c], Yang Yang[g], Lijun Yuan[e], Yan Han**[b] and Guodong Yang*[c]

Table S1. Sequences of aptamers and releasers

**aptamer/ releaser**	**Sequence**
Ldlr aptamer	5'GUCUUUCAGGAAGAUCAUACAUGAGGAUCACCCAUGUCCUUAGCUUCUGCGUACAUGAGGAUCACCCAUGUCUGCAGGACAUGAGGAUCACCCAUGU-3'
Ctrl aptamer	5'GUCUUUCAGGAAGAUCAUACAUGAGGAUCACCCAUGUUUCCGAUCCUCAUACACAUGAGGAUCACCCAUGUCUGCAGGACAUGAGGAUCACCCAUGU-3'
Ldlr releaser 1	5'-CUGCAGGACGCAGAAGCUAAGG-3'
Ldlr releaser 2	5'-UAUGAUCUUCCUGAAAGACAA-3'
Ctrl releaser 1	5'-UUCAUGAGUAUGAGGAUCGGAA-3'
Ctrl releaser 2	5'-UGCAGCUCCUUCAGGGAGUAA-3'

Table S2. Sequences of primers

**qPCR**		
Mouse *Ldlr*	Forward	5'-TGACTCAGACGAACAAGGCTG-3'
	Reverse	5'-ATCTAGGCAATCTCGGTCTCC-3'
Mouse *Gapdh*	Forward	5'-AGGTCGGTGTGAACGGATTTG-3'
	Reverse	5'-TGTAGACCATGTAGTTGAGGTCA-3'
U6	Forward	5'-CTCGCTTCGGCAGCACA-3'
	Reverse	5'-AACGCTTCACGAATTTGCGT-3'
Ldlr releaser 1	Forward	5'-CTGCAGGACGCAGAAGCTAAGG-3'
	Reverse	Provided in the kit
Ldlr releaser 2	Forward	5'-TATGATCTTCCTGAAAGACAA-3'
	Reverse	Provided in the kit
Ctrl releaser 1	Forward	5'-TTCATGAGTATGAGGATCGGAA-3'
	Reverse	Provided in the kit

Ctrl releaser 2	Forward	5'-TGCAGCTCCTTCAGGGAGTAA-3'
	Reverse	Provided in the kit
**Nested PCR**		
External primer of *Ldlr*	Forward	5'-CTCCCAGGATGACTTCCGAT-3'
	Reverse	5'-CGCAGTGCTCCTCATCTGAC-3'
Internal primer of *Ldlr*	Forward	5'-CGACGGGGATGTCGACTGTGTTGA-3'
	Reverse	5'-TCGGCCCTGGCAGTTCTGTG-3'

Table. S3 Sequences of plasmids

**Plasma**	**Sequence**
CD9-MCP	ATGCCGGTCAAAGGAGGTAGCAAGTGCATCAAA TACCTGCTCTTCGGATTTAACTTCATCTTCTGGCT CGCTGGCATTGCAGTGCTTGCTATTGGACTATGG CTCCGATTCGACTCTCAGACCAAGAGCATCTTCG AGCAAGAGAATAACCATTCCAGTTTCTACACAG GAGTGTACATTCTGATTGGAGCCGGGGCCCTCAT GATGCTGGTTGGTTTCCTGGGCTGCTGTGGAGCT GTACAAGAGTCCCAGTGCATGCTGGGATTGTTCT TCGGGTTCCTCTTGGTGATATTCGCCATTGAGAT AGCCGCCGCCGTCTGGGGCTATACCCACAAGGA TGAGGTGATTAAAGAACTCCAGGAGTTTTACAA GGACACCTACCAAAAGTTACGGAGCAAGGATGA ACCCCAGCGGGAAACACTCAAAGCCATCCATAT GGCGTTGGACTGCTGTGGCATAGCTGGTCCTTTG GAGCAGTTTATCTCGGACACCTGCCCCAAGAAA CAGCTTTTGGAAAGTTTCCAGGTTAAGCCCTGCC CTGAAGCCATCAGTGAGGTCTTCAACAACAAGT TCCACATCATTGGAGCAGTGGGTATCGGCATCG CCGTGGTGATGATCTTCGGCATGATCTTCAGCAT GATCCTGTGCTGCGCCATCCGCAGGAGCCGAGA AATGGTCATGGCTTCAAACTTTACTCAGTTCGTG CTCGTGGACAATGGTGGGACAGGGGATGTGACA GTGGCTCCTTCTAATTTCGCTAATGGGGTGGCAG AGTGGATCAGCTCCAACTCACGGAGCCAGGCCT ACAAGGTGACATGCAGCGTCAGGCAGTCTAGTG CCCAGAAGAGAAAGTATACCATCAAGGTGGAGG TCCCCAAAGTGGCTACCCAGACAGTGGGCGGAG TCGAACTGCCTGTCGCCGCTTGGAGGTCCTACCT GAACATGGAGCTCACTATCCCAATTTTCGCTACC AATTCTGACTGTGAACTCATCGTGAAGGCAATG CAGGGGCTCCTCAAAGACGGTAATCCTATCCCTT CCGCCATCGCCGCTAACTCAGGTATCTACTAG
Ldlr	ATGAGCACCGCGGATCTGATGCGTCGCTGGGTC ATCGCCCTGCTCCTGGCTGCTGCCGGAGTTGCAG CAGAAGACTCATGCAGCAGGAACGAGTTCCAGT GTAGAGACGGAAAATGCATCGCTAGCAAGTGGG

	TGTGCGATGGCAGCCCCGAGTGCCCGGATGGCT<br>CCGATGAGTCCCCAGAGACATGCATGTCTGTCA<br>CCTGTCAGTCCAATCAATTCAGCTGTGGAGGCCG<br>TGTCAGCCGATGCATTCCTGACTCCTGGAGATGT<br>GATGGACAGGTAGACTGTGAAAATGACTCAGAC<br>GAACAAGGCTGTCCCCCCAAGACGTGCTCCCAG<br>GATGACTTCCGATGCCAGGATGGCAAGTGCATC<br>TCCCCGCAGTTTGTGTGTGATGGAGACCGAGATT<br>GCCTAGATGGCTCTGATGAGGCCCACTGCCAGG<br>CCACCACTTGTGGCCCCGCCCACTTCCGCTGCAA<br>CTCATCCATATGCATCCCCAGTCTTTGGGCCTGC<br>GACGGGGATGTCGACTGTGTTGACGGCTCCGAT<br>GAGTGGCCACAGAACTGCCAGGGCCGAGACACG<br>GCCTCCAAAGGCGTTAGCAGCCCCTGCTCCTCCC<br>TGGAGTTCCACTGTGGTAGCAGTGAGTGTATCCA<br>TCGCAGCTGGGTCTGTGACGGCGAGGCAGACTG<br>CAAGGACAAGTCAGATGAGGAGCACTGCGCGGT<br>GGCCACCTGCCGACCTGATGAATTCCAGTGTGC<br>AGATGGCTCCTGCATTCACGGTAGCCGCCAGTGT<br>GACCGTGAACATGACTGCAAGGACATGAGCGAC<br>GAGCTCGGCTGCGTCAATGTGACACAGTGTGAT<br>GGCCCCAACAAGTTCAAGTGTCACAGTGGGGAG<br>TGCATCAGCTTGGACAAGGTGTGCGACTCCGCC<br>CGCGACTGCCAGGACTGGTCGGATGAGCCCATC<br>AAGGAGTGCAAGACCAACGAGTGTTTGGACAAC<br>AATGGTGGCTGTTCCCACATCTGCAAGGACCTCA<br>AGATTGGCTCTGAGTGCCTGTGTCCCAGCGGCTT<br>CCGGTTGGTGGACCTCCACAGGTGTGAAGATAT<br>TGACGAGTGTCAGGAGCCAGACACCTGCAGCCA<br>GCTCTGTGTGAACCTGGAAGGCAGCTACAAGTG<br>TGAGTGCCAGGCCGGCTTCCACATGGACCCACA<br>CACCAGGGTCTGCAAGGCTGTGGGCTCCATAGG<br>CTATCTGCTCTTCACCAACCGCCACGAGGTCCGG<br>AAGATGACCCTGGACCGCAGCGAGTACACCAGT<br>CTGCTCCCCAACCTGAAGAATGTGGTGGCTCTCG<br>ACACGGAGGTGACCAACAATAGAATCTACTGGT<br>CCGACCTGTCCCAAAAAAAGATCTACAGCGCCC<br>TGATGGACCAGGCCCCTAACTTGTCCTACGACAC<br>CATCATCAGTGAGGACCTGCATGCCCCTGACGG<br>GCTGGCGGTAGACTGGATCCACCGCAACATCTA<br>CTGGACAGATTCAGTCCCAGGCAGCGTATCTGT<br>GGCTGACACCAAGGGCGTAAAGAGGAGGACACT<br>GTTCCAAGAGGCAGGGTCCAGACCCAGAGCCAT<br>CGTAGTGGACCCTGTGCATGGCTTCATGTACTGG<br>ACAGATTGGGGAACACCCGCCAAGATCAAGAAA<br>GGGGGTTTGAATGGTGTGGACATCCACTCACTG

	GTGACCGAAAACATCCAGTGGCCAAATGGCATC<br>ACACTAGATCTTTCCAGTGGCCGTCTCTATTGGG<br>TTGATTCCAAACTCCACTCTATCTCCAGCATCGA<br>TGTCAATGGGGGCAATCGGAAAACCATTTTGGA<br>GGATGAGAACCGGCTGGCCCACCCCTTCTCCTTG<br>GCCATCTATGAGGACAAAGTGTATTGGACAGAT<br>GTCATAAACGAAGCCATTTTCAGTGCCAATCGA<br>CTCACGGGTTCAGATGTGAATTTGGTGGCTGAA<br>AACCTCTTGTCCCCGGAGGACATTGTCCTGTTCC<br>ACAAGGTCACACAGCCTAGAGAAGTCGACACTG<br>TACTGACCACCCAGGGGACATCCGCCGTCCGGC<br>CTGTGGTCACCGCATCAGCTACCAGGCCACCGA<br>AGCACAGTGAGGATCTCTCAGCTCCCAGTACTCC<br>TAGGCAGCCTGTGGACACCCCAGGGCTCAGCAC<br>AGTGGCGTCAGTGACAGTGTCCCACCAAGTCCA<br>GGGTGACATGGCTGGCAGAGGGAATGAGGAGC<br>AGCCACATGGTATGAGGTTCCTGTCCATCTTCTT<br>CCCTATTGCACTGGTTGCCCTCCTTGTCCTTGGG<br>GCCGTCCTGCTGTGGAGGAACTGGCGGCTGAAG<br>AACATCAACAGCATAAACTTTGACAACCCAGTC<br>TACCAGAAGACCACAGAGGACGAGCTCCACATT<br>TGCCGAAGCCAGGATGGCTATACCTACCCCTCA<br>AGACAGATGGTCAGCCTGGAGGACGATGTGGCA<br>TGA
Ldlr-MS2	ATGAGCACCGCGGATCTGATGCGTCGCTGGGTC<br>ATCGCCCTGCTCCTGGCTGCTGCCGGAGTTGCAG<br>CAGAAGACTCATGCAGCAGGAACGAGTTCCAGT<br>GTAGAGACGGAAAATGCATCGCTAGCAAGTGGG<br>TGTGCGATGGCAGCCCCGAGTGCCCGGATGGCT<br>CCGATGAGTCCCCAGAGACATGCATGTCTGTCA<br>CCTGTCAGTCCAATCAATTCAGCTGTGGAGGCCG<br>TGTCAGCCGATGCATTCCTGACTCCTGGAGATGT<br>GATGGACAGGTAGACTGTGAAAATGACTCAGAC<br>GAACAAGGCTGTCCCCCCAAGACGTGCTCCCAG<br>GATGACTTCCGATGCCAGGATGGCAAGTGCATC<br>TCCCCGCAGTTTGTGTGTGATGGAGACCGAGATT<br>GCCTAGATGGCTCTGATGAGGCCCACTGCCAGG<br>CCACCACTTGTGGCCCCGCCCACTTCCGCTGCAA<br>CTCATCCATATGCATCCCCAGTCTTTGGGCCTGC<br>GACGGGGATGTCGACTGTGTTGACGGCTCCGAT<br>GAGTGGCCACAGAACTGCCAGGGCCGAGACACG<br>GCCTCCAAAGGCGTTAGCAGCCCCTGCTCCTCCC<br>TGGAGTTCCACTGTGGTAGCAGTGAGTGTATCCA<br>TCGCAGCTGGGTCTGTGACGGCGAGGCAGACTG<br>CAAGGACAAGTCAGATGAGGAGCACTGCGCGGT<br>GGCCACCTGCCGACCTGATGAATTCCAGTGTGC

	AGATGGCTCCTGCATTCACGGTAGCCGCCAGTGT<br>GACCGTGAACATGACTGCAAGGACATGAGCGAC<br>GAGCTCGGCTGCGTCAATGTGACACAGTGTGAT<br>GGCCCCAACAAGTTCAAGTGTCACAGTGGGGAG<br>TGCATCAGCTTGGACAAGGTGTGCGACTCCGCC<br>CGCGACTGCCAGGACTGGTCGGATGAGCCCATC<br>AAGGAGTGCAAGACCAACGAGTGTTTGGACAAC<br>AATGGTGGCTGTTCCCACATCTGCAAGGACCTCA<br>AGATTGGCTCTGAGTGCCTGTGTCCCAGCGGCTT<br>CCGGTTGGTGGACCTCCACAGGTGTGAAGATAT<br>TGACGAGTGTCAGGAGCCAGACACCTGCAGCCA<br>GCTCTGTGTGAACCTGGAAGGCAGCTACAAGTG<br>TGAGTGCCAGGCCGGCTTCCACATGGACCCACA<br>CACCAGGGTCTGCAAGGCTGTGGGCTCCATAGG<br>CTATCTGCTCTTCACCAACCGCCACGAGGTCCGG<br>AAGATGACCCTGGACCGCAGCGAGTACACCAGT<br>CTGCTCCCCAACCTGAAGAATGTGGTGGCTCTCG<br>ACACGGAGGTGACCAACAATAGAATCTACTGGT<br>CCGACCTGTCCCAAAAAAAGATCTACAGCGCCC<br>TGATGGACCAGGCCCCTAACTTGTCCTACGACAC<br>CATCATCAGTGAGGACCTGCATGCCCCTGACGG<br>GCTGGCGGTAGACTGGATCCACCGCAACATCTA<br>CTGGACAGATTCAGTCCCAGGCAGCGTATCTGT<br>GGCTGACACCAAGGGCGTAAAGAGGAGGACACT<br>GTTCCAAGAGGCAGGGTCCAGACCCAGAGCCAT<br>CGTAGTGGACCCTGTGCATGGCTTCATGTACTGG<br>ACAGATTGGGGAACACCCGCCAAGATCAAGAAA<br>GGGGGTTTGAATGGTGTGGACATCCACTCACTG<br>GTGACCGAAAACATCCAGTGGCCAAATGGCATC<br>ACACTAGATCTTTCCAGTGGCCGTCTCTATTGGG<br>TTGATTCCAAACTCCACTCTATCTCCAGCATCGA<br>TGTCAATGGGGGCAATCGGAAAACCATTTTGGA<br>GGATGAGAACCGGCTGGCCCACCCCTTCTCCTTG<br>GCCATCTATGAGGACAAAGTGTATTGGACAGAT<br>GTCATAAACGAAGCCATTTTCAGTGCCAATCGA<br>CTCACGGGTTCAGATGTGAATTTGGTGGCTGAA<br>AACCTCTTGTCCCCGGAGGACATTGTCCTGTTCC<br>ACAAGGTCACACAGCCTAGAGAAGTCGACACTG<br>TACTGACCACCCAGGGGACATCCGCCGTCCGGC<br>CTGTGGTCACCGCATCAGCTACCAGGCCACCGA<br>AGCACAGTGAGGATCTCTCAGCTCCCAGTACTCC<br>TAGGCAGCCTGTGGACACCCCAGGGCTCAGCAC<br>AGTGGCGTCAGTGACAGTGTCCCACCAAGTCCA<br>GGGTGACATGGCTGGCAGAGGGAATGAGGAGC<br>AGCCACATGGTATGAGGTTCCTGTCCATCTTCTT<br>CCCTATTGCACTGGTTGCCCTCCTTGTCCTTGGG

	GCCGTCCTGCTGTGGAGGAACTGGCGGCTGAAG<br>AACATCAACAGCATAAACTTTGACAACCCAGTC<br>TACCAGAAGACCACAGAGGACGAGCTCCACATT<br>TGCCGAAGCCAGGATGGCTATACCTACCCCTCA<br>AGACAGATGGTCAGCCTGGAGGACGATGTGGCA<br>TGAGGTGCCCGCTTCCACTCCCCACCCCGATATC<br>CGCAGCTAGGCCAACATGAGGATCACCCATGTC<br>TGCAGGGCCTAGCGGTGCCCGCTTCCACTCCCCA<br>CCCCGATATCCGCAGCTAGGCCAACATGAGGAT<br>CACCCATGTCTGCAGGGCCTAGCGGTGCCCGCTT<br>CCACTCCCCACCCCGATATCCGCAGCTAGGCCA<br>ACATGAGGATCACCCATGTCTGCAGGGCCTAGC

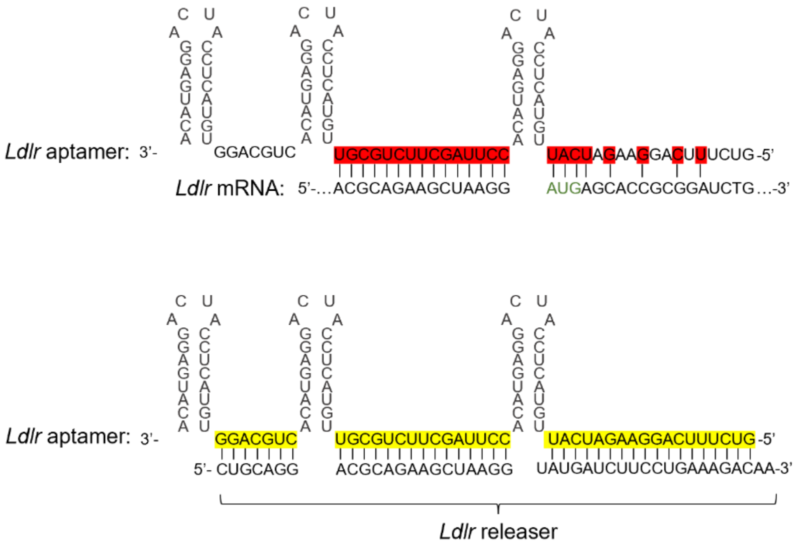

Figure S1. Design of *Ldlr* aptamer and *Ldlr* releaser: *Ldlr* aptamer with 23 bases pairing with *Ldlr* mRNA near its initiation codon. The pairing bases of *Ldlr* aptamer are denoted by red. *Ldlr* releaser contained 22 and 18 bases pairing with *Ldlr* aptamer respectively. The pairing bases of *Ldlr* aptamer are denoted by yellow.

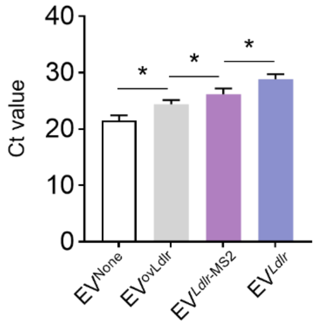

Figure S2. qPCR analysis of Ct value of GAPDH in EVs**:** About 100 μg EVs, collected from cells without transfection (EV^{None}), transfected with *Ldlr*-expressing vector (EV^{ovLdlr}), co-transfected with *Ldlr*-MS2 expressing vector and CD9-MCP vector ($EV^{Ldlr\text{-}MS2}$), or co-transfected with *Ldlr*-expressing vector, *Ldlr* aptamer, CD9-MCP vector (EV^{Ldlr}) were used for RNA isolation, followed by qPCR analysis of GAPDH abundance. Data are presented as mean±SEM of 3 independent

experiments. *, $P<0.05$ by One-way-ANOVA.

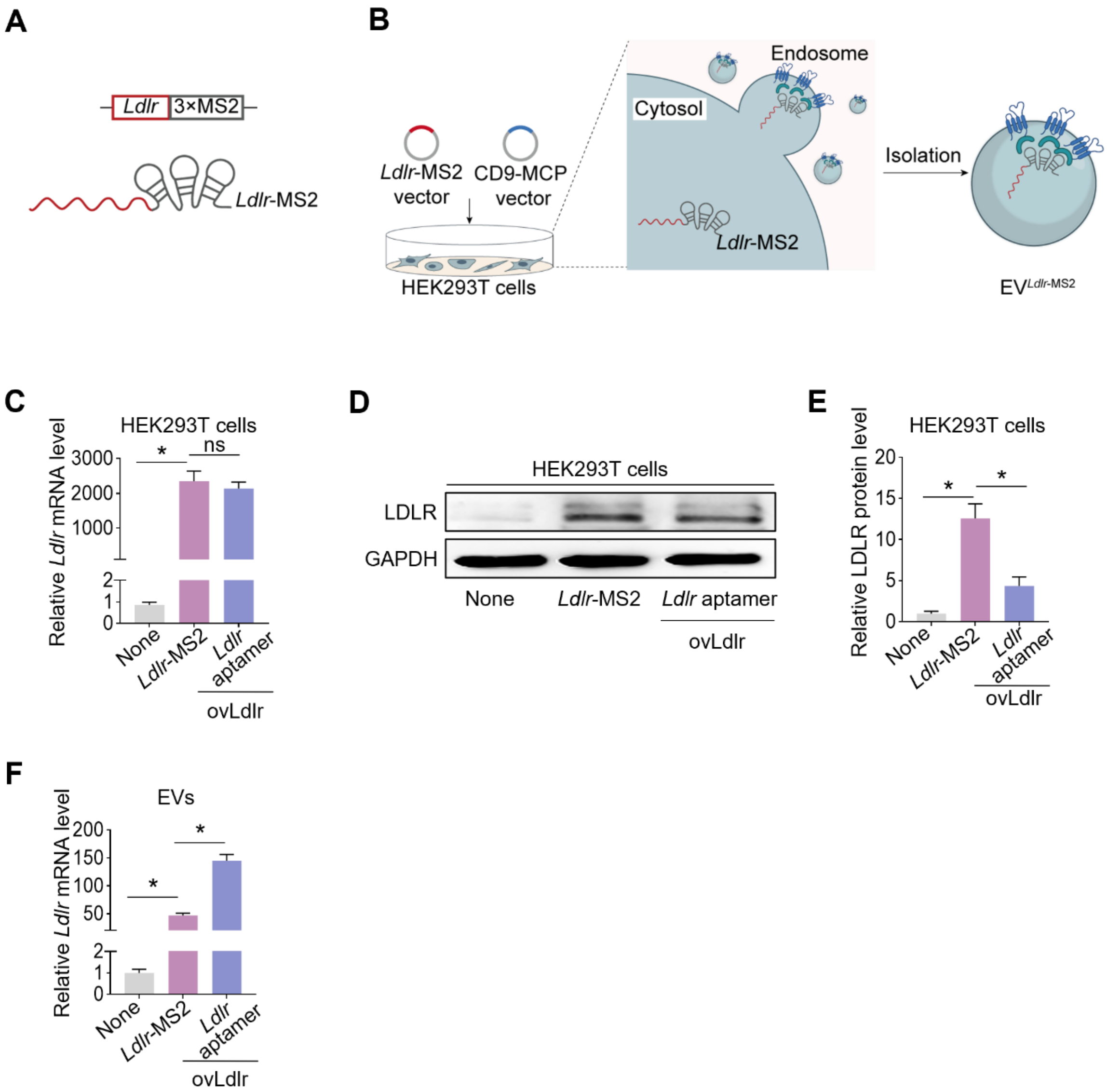

Figure S3. Construction of EV$^{Ldlr\text{-MS2}}$ and EV loading efficiency of *Ldlr* mRNA: (A) Cloning of *Ldlr*-MS2-expressing vector. (B) Schematic illustration of *Ldlr*-MS2 encapsulated into engineered CD9-MCP EVs. *Ldlr*-MS2-expressing and CD9-MCP-expressing vectors were simultaneously transfected into HEK293T cells. *Ldlr* mRNA linked with MS2 stem loops was expressed by *Ldlr*-MS2-expressing vector and then enriched into CD9-MCP-engineered EVs through the interaction of MS2-MCP. (C) qPCR analysis of *Ldlr* mRNA in HEK293T cells treated as indicated. (D)

Western blot analysis of LDLR protein in HEK293 cells treated as indicated. Representative data of 3 independent experiments. (E) Quantification of Western blot bands by densitometry. (F) qPCR analysis of *Ldlr* mRNA in HEK293T cells derived EVs as indicated. Data are presented as mean±SEM of 3 independent experiments. *, $P<0.05$ by t-test. ns, no significance.

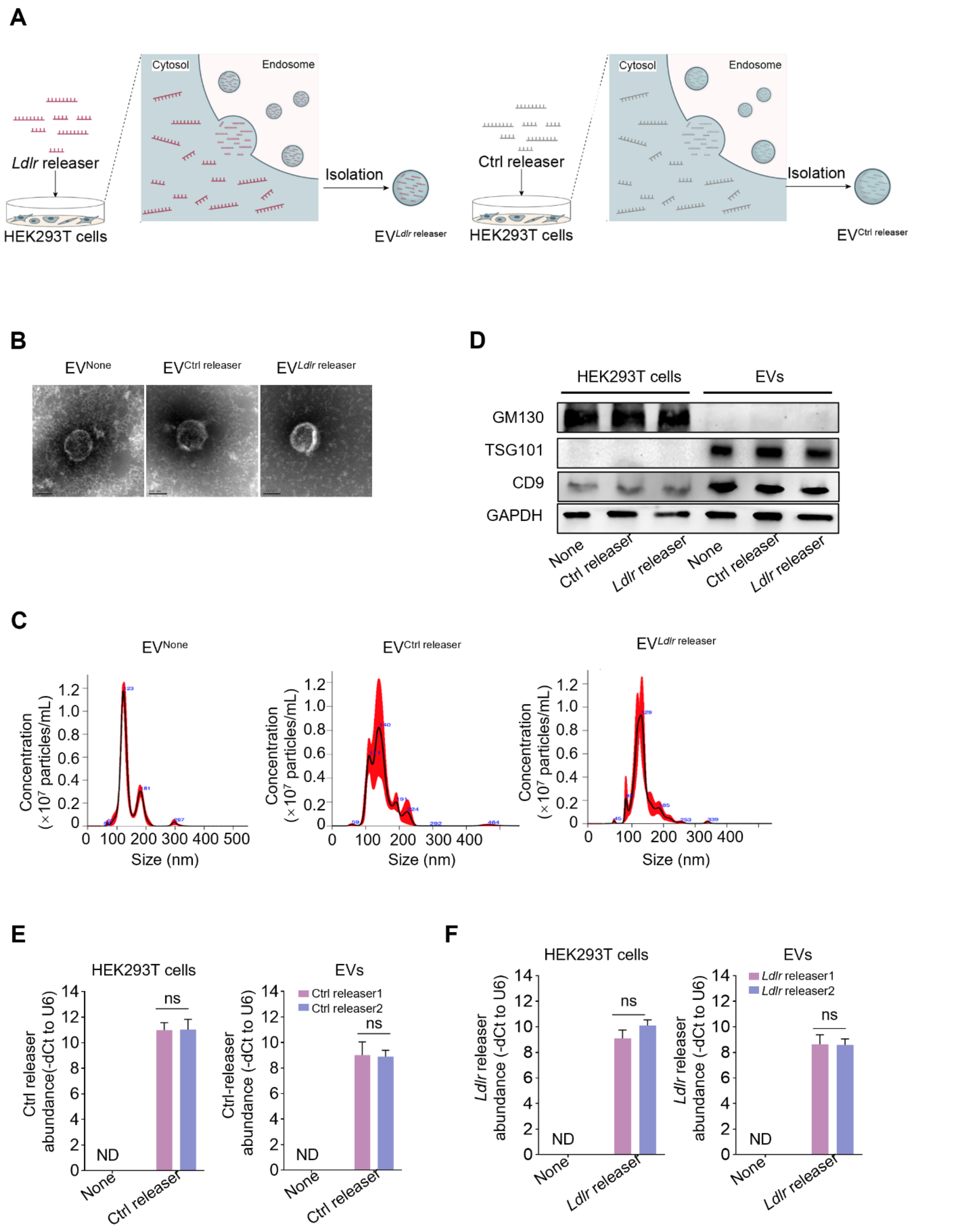

Figure S4. Construction and characterization of EV$^{Ldlr\ releaser}$: (A) Schematic

illustration of Exo$^{Ldlr\text{ releaser}}$ preparation and isolation (B) Representative TEM images of indicated exosomes. Scale bar=100 nm (C) Size distribution of indicated exosomes (D) Western blot analysis of the inclusive exosome markers TSG101, CD63, and the exclusive marker GM130. Representative data of 3 independent experiments. (E) qPCR analysis of Ctrl releaser in HEK293T cells treated as indicated and derived EVs. U6 served as an internal control. (F) qPCR analysis of *Ldlr* releaser in both HEK293T cells and derived exosomes treated as indicated. U6 served as an internal control. ND, not determined as Ct value greater than 38. Data are presented as mean±SEM of 3 independent experiments.

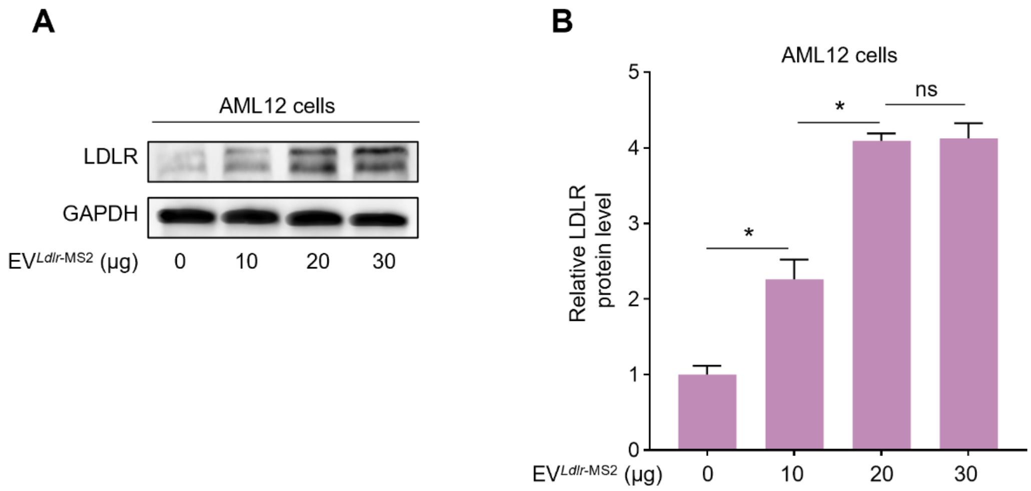

Figure S5. LDLR expression in AML12 cells co-cultured with increasing amounts of EV$^{Ldlr\text{-MS2}}$: (A) Western blot analysis of LDLR protein expression in AML12 cells co-cultured with increasing amount of EV$^{Ldlr\text{-MS2}}$ as indicated. Representative data of 3 independent experiments. (B) Quantification of Western blot bands by densitometry. Data are presented as mean±SEM of 3 independent experiments. *$P<0.05$ by one-way ANOVA. ns, no significance.

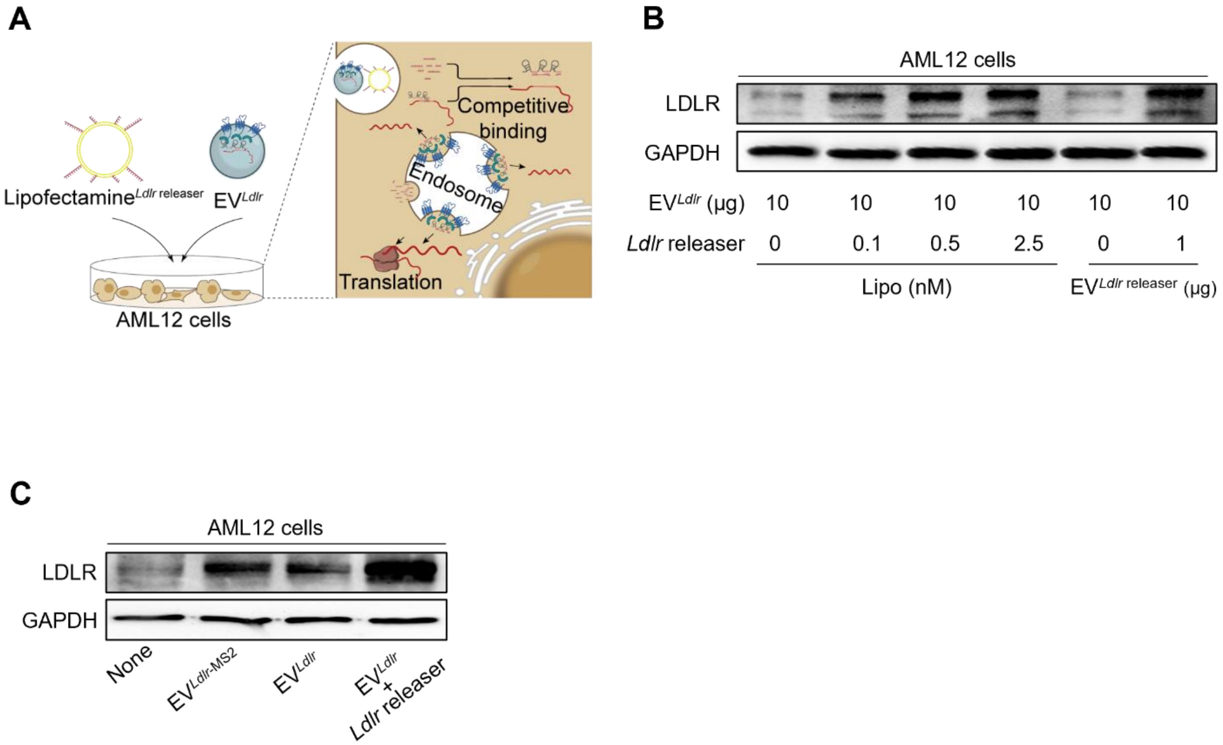

Figure S6. Competitive binding of *Ldlr* releaser with *Ldlr* aptamer: (A) Schematic illustration of *Ldlr* mRNA in Exo^{Ldlr} released by AML12 cells transfected with *Ldlr* releaser and incubated with EV^{Ldlr}. *Ldlr* releaser competitively binds to *Ldlr* aptamer resulting in *Ldlr* mRNA release into the cytosol, which is translated into protein. (B) Western blot analysis of LDLR protein expression in AML12 cells after transfection with different doses of *Ldlr* releaser via lipofectamine or EV, followed by incubation with 10 μg Exo^{Ldlr}. (C) Western blot analysis of LDLR protein expression in AML12 cells after co-culturing with EV^{releaser}/EV^{Ldlr} or Exo$^{Ldlr\text{-MS2}}$ as indicated. GAPDH served as the loading control. Representative data of 3 independent experiments.

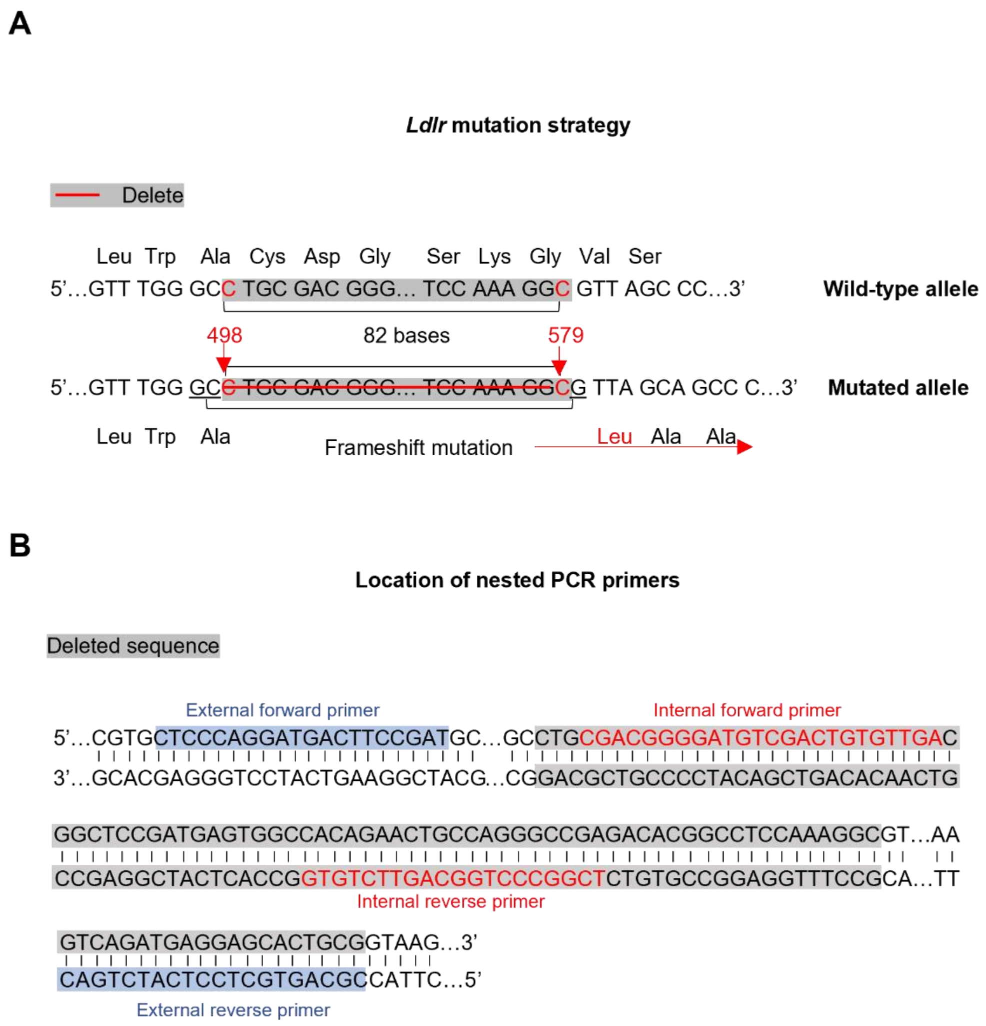

Figure S7. Illustration of *Ldlr* gene deletion strategy and primer design: (A) *Ldlr* gene deletion strategy. In the knockout mice, an 82 bp sequence was deleted, resulting in a frameshift. (B) Schematic representation of the location of primers for nested PCR and qPCR.

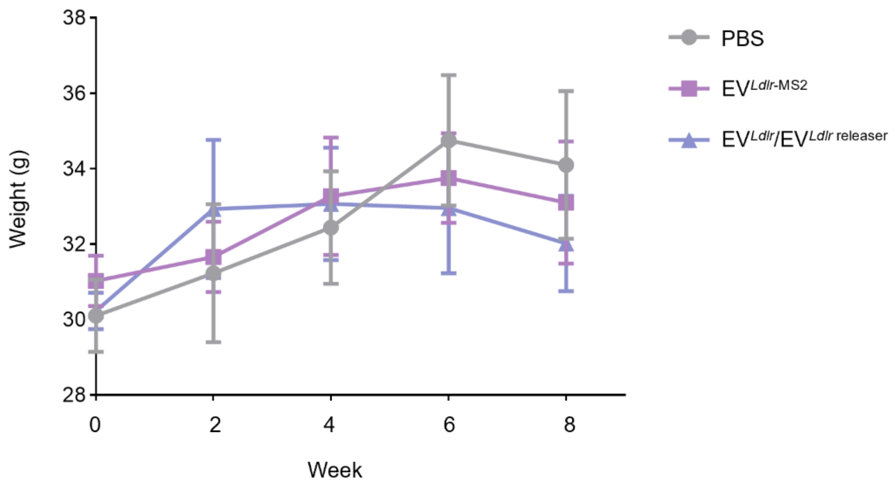

Figure S8. Body weights of mice with different treatments. No significant differences in body weights were observed. Data are presented as mean±SEM, n=6. No significant difference was detected by two-way ANOVA.
